# Rudhira/BCAS3 is essential for mouse development and cardiovascular patterning

**DOI:** 10.1038/s41598-018-24014-w

**Published:** 2018-04-04

**Authors:** Ronak Shetty, Divyesh Joshi, Mamta Jain, Madavan Vasudevan, Jasper Chrysolite Paul, Ganesh Bhat, Poulomi Banerjee, Takaya Abe, Hiroshi Kiyonari, K. VijayRaghavan, Maneesha S. Inamdar

**Affiliations:** 1 Jawaharlal Nehru Centre for Advanced Scientific Research, Bangalore, India; 2 RIKEN Center for Life Science Technologies, Kobe, Japan; 3 Bionivid, Kasturi Nagar, Bangalore, India; 4 National Centre for Biological Sciences, Bangalore, India; 5 Institute for Stem Cell biology and Regenerative Medicine (inStem), Bangalore, India

## Abstract

Rudhira/Breast Carcinoma Amplified Sequence 3 (BCAS3) is a cytoskeletal protein that promotes directional cell migration and angiogenesis *in vitro* and is implicated in human carcinomas and coronary artery disease. To study the role of Rudhira during development *in vivo*, we generated the first knockout mouse for *rudhira* and show that Rudhira is essential for mouse development. *Rudhira* null embryos die at embryonic day (E) 9.5 accompanied by severe vascular patterning defects in embryonic and extra-embryonic tissues. To identify the molecular processes downstream of *rudhira*, we analyzed the transcriptome of intact knockout yolk sacs. Genome-wide transcriptome analysis showed that Rudhira functions in angiogenesis and its related processes such as cell adhesion, extracellular matrix organization, peptidase activity and TGFβ signaling. Since Rudhira is also expressed in endothelial cells (ECs), we further generated Tie2Cre-mediated endothelial knockout (CKO) of *rudhira*. CKO embryos survive to E11.5 and similar to the global knockout, display gross vascular patterning defects, showing that endothelial Rudhira is vital for development. Further, Rudhira knockdown ECs in culture fail to sprout in a spheroid-sprouting assay, strongly supporting its role in vascular patterning. Our study identifies an essential role for Rudhira in blood vessel remodeling and provides a mouse model for cardiovascular development.

## Introduction

The cytoskeleton is composed of microtubules, microfilaments and intermediate filaments that regulate each other and are intimately linked in several normal and diseased contexts^1^. Cytoskeletal reorganization is a major requirement in processes that require change in cell adhesion and migration status^2–4^. This is accompanied by change in cell shape and gene expression through a complex network of signaling pathways by influencing transcription factor activity or otherwise. Homozygous mutants of tubulins and microtubule-associated proteins (MAPs) show pre-gastrulation lethality on the one hand or display mild developmental phenotypes on the other^5–7^. However, the functions of a large number of cytoskeletal proteins remain to be elucidated.

Rudhira is a cytoskeletal WD40 domain containing protein that binds microtubules and promotes directional cell migration *in vitro* by activating Cdc42 for actin reorganization and filopodial extension^8^. Rudhira knockdown leads to random and retarded cell migration *in vitro* whereas overexpression promotes migration in non-motile cells. Rudhira is expressed in mouse embryonic stem cells, primitive erythropoiesis and vascular precursors, endothelial cells (ECs), in human vascular development in an *in vitro* embryoid body model and in malignant tumors and blood vessels^8–10^. Human Rudhira/Breast Cancer Amplified Sequence 3 (BCAS3) has recently been associated with coronary artery disease^11^. To elucidate the normal role of Rudhira *in vivo* we generated *rudhira* mutant mice using Cre-loxP mediated deletion and analyzed the consequence of *rudhira* deficiency *in vivo*.

We report for the first time that *rudhira* deletion results in mid-gestation lethality with aberrant cardiovascular patterning. Rudhira deletion causes aberrant gene expression as seen by yolk sac transcriptome studies. We show that endothelial Rudhira is essential for angiogenesis and vascular remodeling during development.

## Results

### *Rudhira* is vital for embryonic development

**Figure 1:**
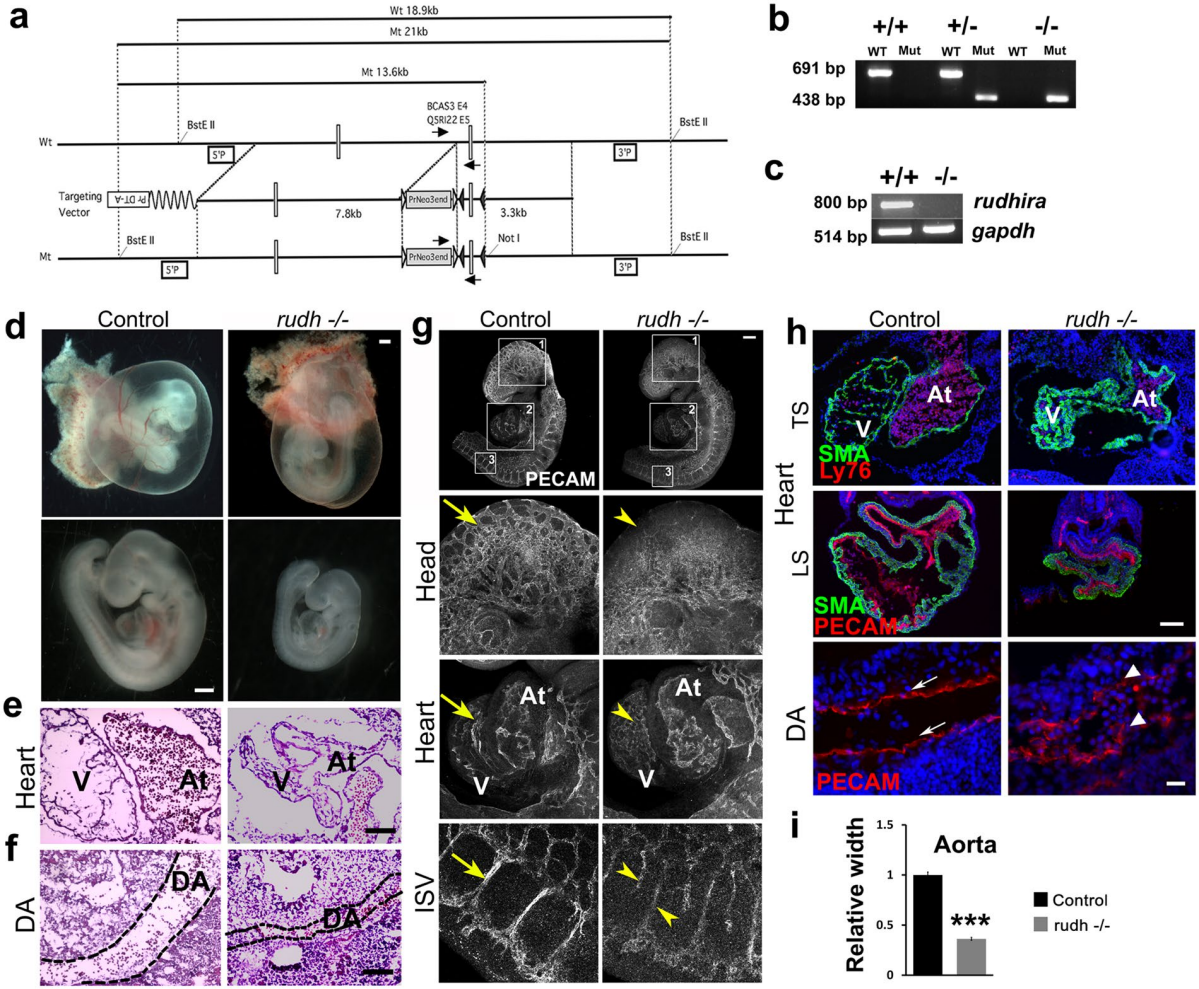
Rudhira is essential for development and cardio-vascular patterning. (**a**) Schematic showing strategy for generation of floxed allele of *rudhira* at exon 6. Rectangles: exons; black and white triangles: *loxP* and *frt* sequences respectively; 5’P and 3’P: probes for Southern blot analyses; arrows: genotyping primers. (**b**) PCR analysis showing genotype of control (+/+), heterozygous knock-out (+/−) and homozygous knock-out (−/−) embryos. (**c**) RT-PCR analysis showing *rudhira* mRNA expression in control (+/+) and homozygous knock-out *rudh*^*−/−*^ (*rudhfl/fl;CMVCre+*) (−/−) embryos. *GAPDH*: loading control. (**d**–**i**) Analysis of control and knockout embryos at E9.5. (**d**) Unstained embryos, (**e**,**f**) Histological analysis comparing heart and dorsal aorta (DA). (**g**) Whole mount PECAM staining in control and *rudh*^−/−^ embryos as indicated, with magnified view of head, heart and intersomitic vessels. (**h**) Immunostaining analysis of heart and dorsal aorta (DA) using myocardial marker SMA, primitive erythroid marker Ly76 and vascular markers PECAM. Arrows: normal vascular patterning; arrowheads: irregular and discontinuous vasculature. N = 5 embryos per genotype. (**i**) Graph shows quantitation of dorsal aorta width in the thoracic region. TS: Transverse section, LS: Lateral section, V: Ventricle, At: Atrium. Black dotted lines mark the boundary of DA. Scale bar: (**d**) 500 μm; (**e**,**f**) 100 μm; (**g**) 500 µm; (**h**) E9.5 heart: 100 μm, DA: 20 μm. The observed chi-square values for two degrees of freedom were 9.18 (E8.5), 13.08 (E9.5), 23.42 (E10.5), 42.96 (E11.5) and 37.5 (Postnatal).

Rudhira is expressed primarily in early embryonic vascular development and neo-angiogenesis, but its role *in vivo* is not known. Hence we generated *rudhira* floxed mice (Fig. 1a and Fig. S1a) and crossed them to *CMV-Cre* for ubiquitous deletion (*rudhira*^*flox/flox*^; *CMVCre*^+^ abbreviated to *rudh*^*−/−*^) (see Materials and methods and Fig. S1a). While heterozygotes were viable, ubiquitous deletion of *rudhira* gave no live homozygous pups (Table S1a). This indicates that deletion of *rudhira* causes recessive embryonic lethality. Analysis of embryos from E8.5 to E11.5 showed a reduced number of homozygous mutant embryos (Table S1a) as identified by genotyping (Fig. 1b), transcript (Fig. 1c) and protein (Fig. S1b,c) expression, suggesting that lethality occurred between E9.0 and E10. Chi-square test showed significantly reduced frequency of knockout embryos from E8.5 onwards, as compared to the expected.

Since *rudhira* expression may be transient or undetectable in some migrating cells we also analyzed the effect of globally deleted *rudhira* (*rudh*^−/−^) on development from E7.5 onwards, a stage before *rudhira* expression is detectable^10^. At E7.5 *rudhira* mutant embryos were indistinguishable from littermate control with respect to morphology as well as primitive streak formation as seen by Brachyury expression (Fig. S1d). However, at E8.5, the mutant embryos showed unpatterned dorsal aorta as detected by Flk1 staining (Fig. S1e-e’). By E9.5, mutant embryos were often growth retarded (20/51 = 39.2%) (Fig. 1d and Fig. S1f) with defects including reduced somite number (19 ± 2 at E9.5 in growth retarded mutants compared to 25 ± 2 in controls; n = 10). This suggests that Rudhira may be essential for multiple developmental processes and hence its depletion leads to embryonic lethality.

### Rudhira plays a key role in cardiovascular development and tissue patterning

In mouse development Rudhira is known to have restricted expression during vasculogenesis and primitive erythropoiesis^10^. Hence, we reasoned that cardiovascular defects could be one major cause of growth retardation and lethality seen in *rudhira* null embryos. To investigate this further, we analyzed the effect of *rudhira* deletion on cardiac and vascular patterning (Fig. 1e–i). Whole mount immunostaining of *rudh*^−/−^ embryos with anti-PECAM1 antibodies showed striking defects in the morphology and vasculature of the head and heart in all mutant embryos analyzed at E9.5 even if they were not developmentally delayed (Fig. 1g). While control embryos had a well formed vascular network comprising major vessels giving rise to intricate secondary and tertiary branches (Fig. 1g, arrows), *rudhira* mutants showed completely disorganized head vasculature with defective vessel sprouting, reduced capillaries and impaired branching of intersomitic vessels (ISVs) that failed to sprout into fine capillaries (Fig. 1g arrowheads). Histological analysis (Fig. 1e,f) as well as immunostaining for cardiovascular markers (Fig. 1h) showed that *rudh*^*−/−*^ embryos had collapsed, smaller heart chambers, reduced endocardium development and a fused atrio-ventricular canal. Dorsal aorta was discontinuous with a pronounced decrease in the lumen and intersomitic vessels were improperly patterned (Fig. 1f–i). The endothelial lining was disorganized in all tissues and ECs seemed to have impaired or random migration and were unable to form organized vessels (Fig. 1g,h). These observations show that Rudhira is essential for development and its loss leads to defects in cardiac and vascular patterning.

### Rudhira also functions in extraembryonic vascular development

**Figure 2:**
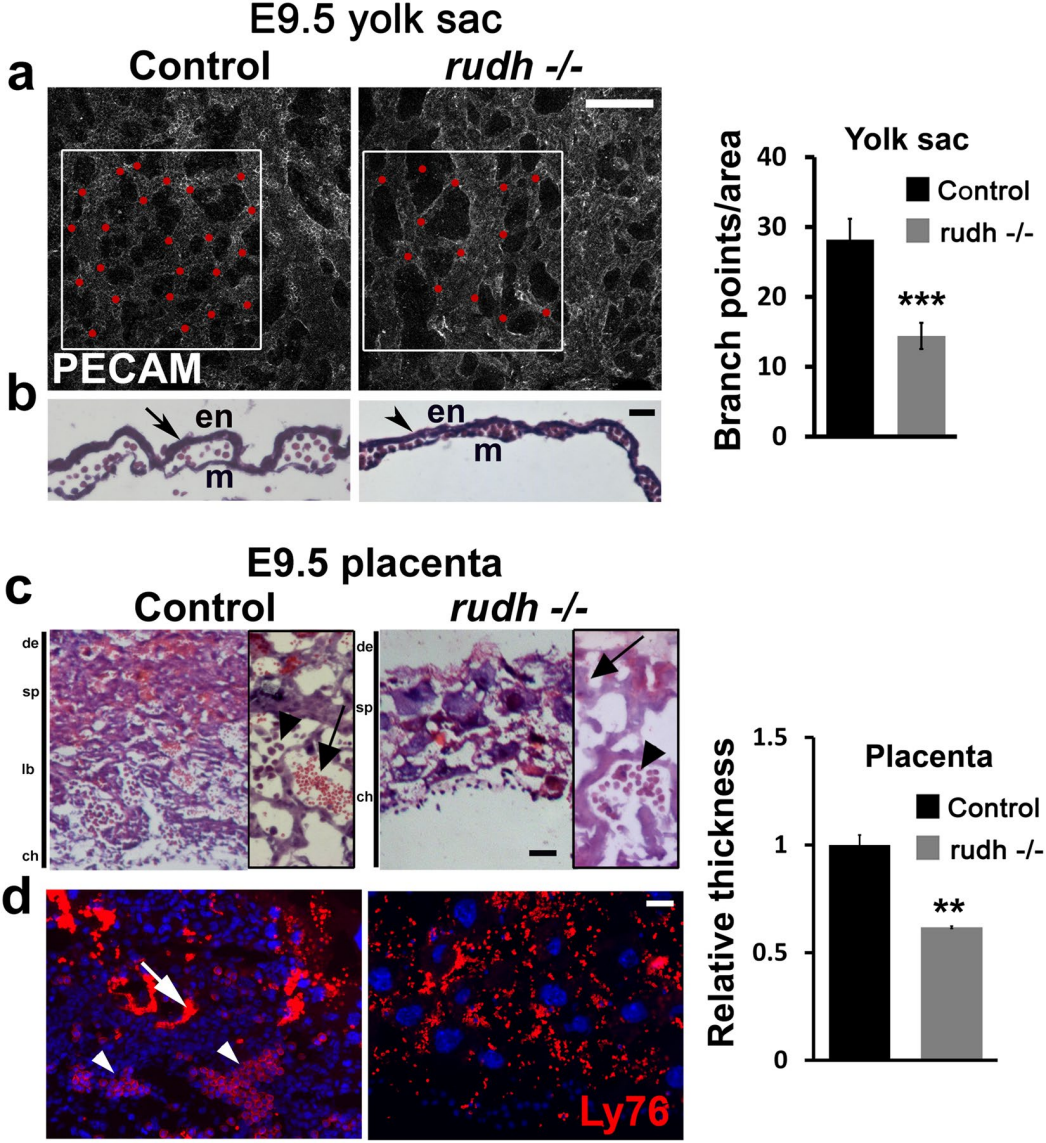
Rudhira is required for extra-embryonic vascular patterning. (**a**) Morphology of whole mount PECAM/CD31 stained yolk sac Red dots: branch points. Graph shows quantitation of branch points (N = 5 yolk sacs). (**b**) hematoxylin-eosin stained yolk sac sections. en: endoderm, m: mesoderm. (**c**,**d**) Placental morphology seen by histological analysis (**c**) or immunofluorescence staining (**d**) in sections of control and *rudh*^*−/−*^ as indicated. Boxed areas (in c) show magnified images of respective placenta. Immunostaining shows fetal and maternal blood cell pockets marked by Ly76. Nuclei are marked by DAPI (Blue). Arrows: maternal blood cells; arrowheads: fetal blood cells. de: decidua, sp: spongiotrophoblast, lb: labyrinth, ch: chorion. Graph shows quantitation of placental thickness. Results shown are a representative of at least three independent experiments with at least three biological replicates each. Statistical analysis was carried out using one-way ANOVA. Scale bar: (**a**,**b**) 200 µm; (**c**,**d**) 100 µm. *p < 0.05, **p < 0.01, ***p < 0.001.

Impaired development and embryonic lethality between E8.5-E11.5 is often the result of aberrant and functionally impaired extra-embryonic vasculature^12^. Moreover, Rudhira is strongly expressed in the yolk sac vasculature^10^. Hence we analyzed extraembryonic structures of mutant embryos, such as yolk sac and placenta, which connect the maternal and fetal vasculature. Mutant yolk sacs were pale and had few major blood vessels (Fig. 1d). Immunostaining for the blood vessel marker PECAM showed that *rudh*^*−/−*^ yolk sac vessels were irregular and fused, unlike the finely patterned honey-comb like vascular network seen in control littermates (Figs 2a, S2). Thus mutants could form a primitive vascular plexus which, however, did not undergo angiogenic remodeling. Histological analyses of yolk sac showed congested capillaries lined by thinner endothelium (Fig. 2b, arrowhead) as compared to controls (Fig. 2b, arrow). These results indicate that *rudhira* is essential for remodeling the yolk sac vascular network. So we reasoned that aberrant vascular remodeling in *rudhira* mutants is likely the primary cause of death.

Placental circulation is vital for nourishment and development of the embryo. Improper development of the labyrinth, the feto-maternal interface, results in poor fetal invasion and causes growth retardation^13^. Morphological analyses showed that *rudhira* null embryos have a smaller placenta with abnormal histology as compared to controls (Fig. 2c,d). Control placenta showed a distinct chorionic plate, labyrinth, spongiotrophoblast and decidual layers. *Rudh*^*−/−*^ placenta lacked stratified layers with a greatly reduced labyrinth and chorionic plate composed mostly of trophoblast giant cells. Fetal blood vessels could not invade into the placenta of *rudh*^*−/−*^ and contained fewer Ly76+ fetal erythrocytes as compared to controls where maternal (arrows) and fetal (arrowheads) blood cell pockets co-existed (Fig. 2c,d). Taken together, these findings suggest that Rudhira is essential for fetal vessel invasion into the developing placenta. Further, growth retardation in *rudh*^*−/−*^ embryos is likely a result of defective placental circulation.

### Rudhira controls the angiogenesis gene regulatory network

**Figure 3:**
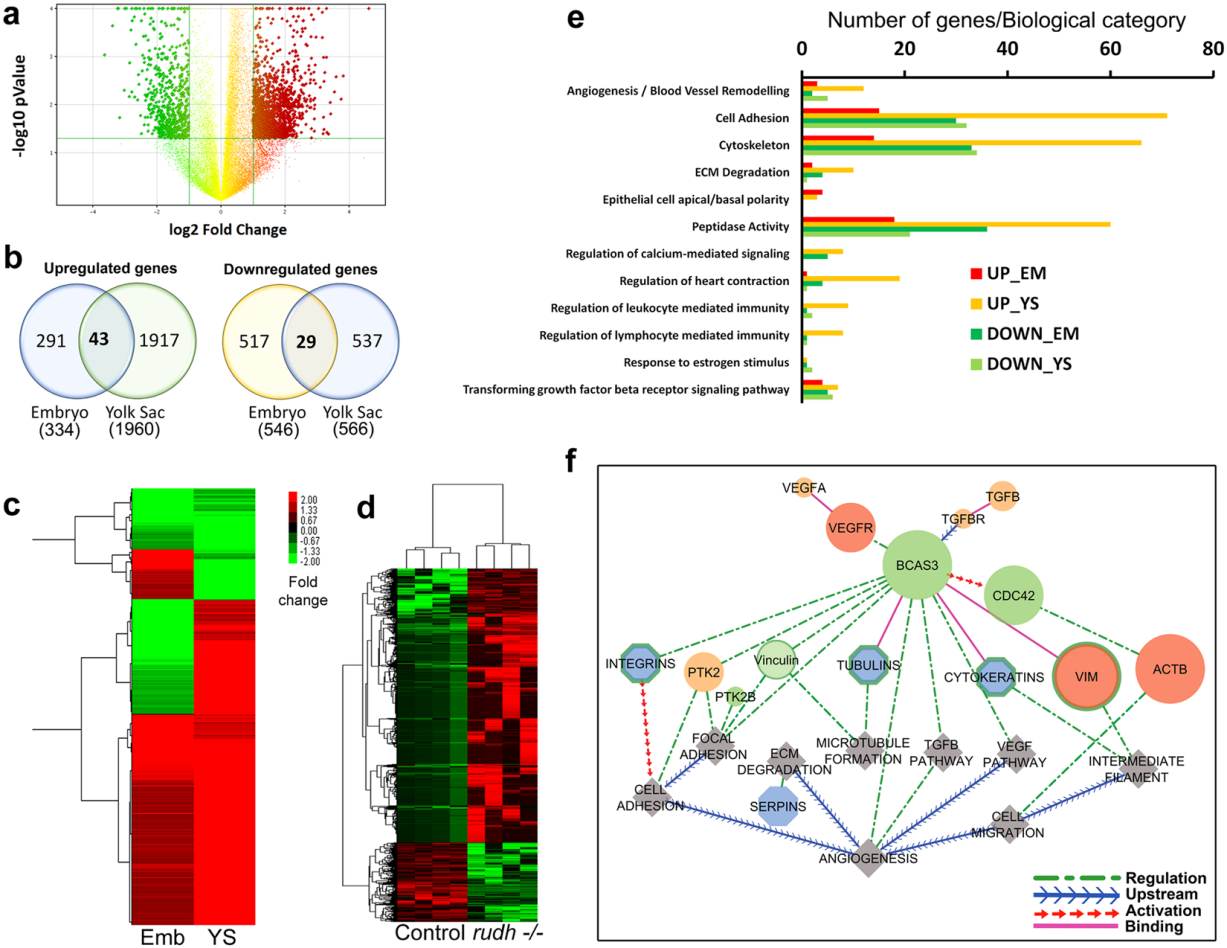
Transcriptome analysis of *rudhira* knockout. (**a**) Representation of differentially expressed genes in *rudh*^*−/−*^ by volcano plot. (**b**) Venn diagram showing number of genes (unique probe sets) dysregulated in embryo and yolk sac upon *rudhira* deletion. (**c**) Unsupervised hierarchical clustering of differentially expressed gene changes between control and *rudh*^*−/−*^ embryo (Emb) and yolk sac (YS). Green: low expression red: high expression. Genes with the most similar expression profiles clustered together with the shortest branches. Dendrogram illustrates their relationship. (**d**) Unsupervised hierarchical clustering of differentially expressed gene changes in *rudh*^*−/−*^ yolk sac compared to controls. Each row represents a gene, and column represents the tissue. (**e**) Histogram showing significantly enriched Gene Ontology (GO) and Pathways (p <  = 0.05) harboring differentially expressed genes in the embryo and yolk sac upon *rudhira* deletion. (**f**) Model depicting *rudhira/BCAS3* gene regulatory pathway visualized with CytoscapeV8.0.

**Figure 4:**
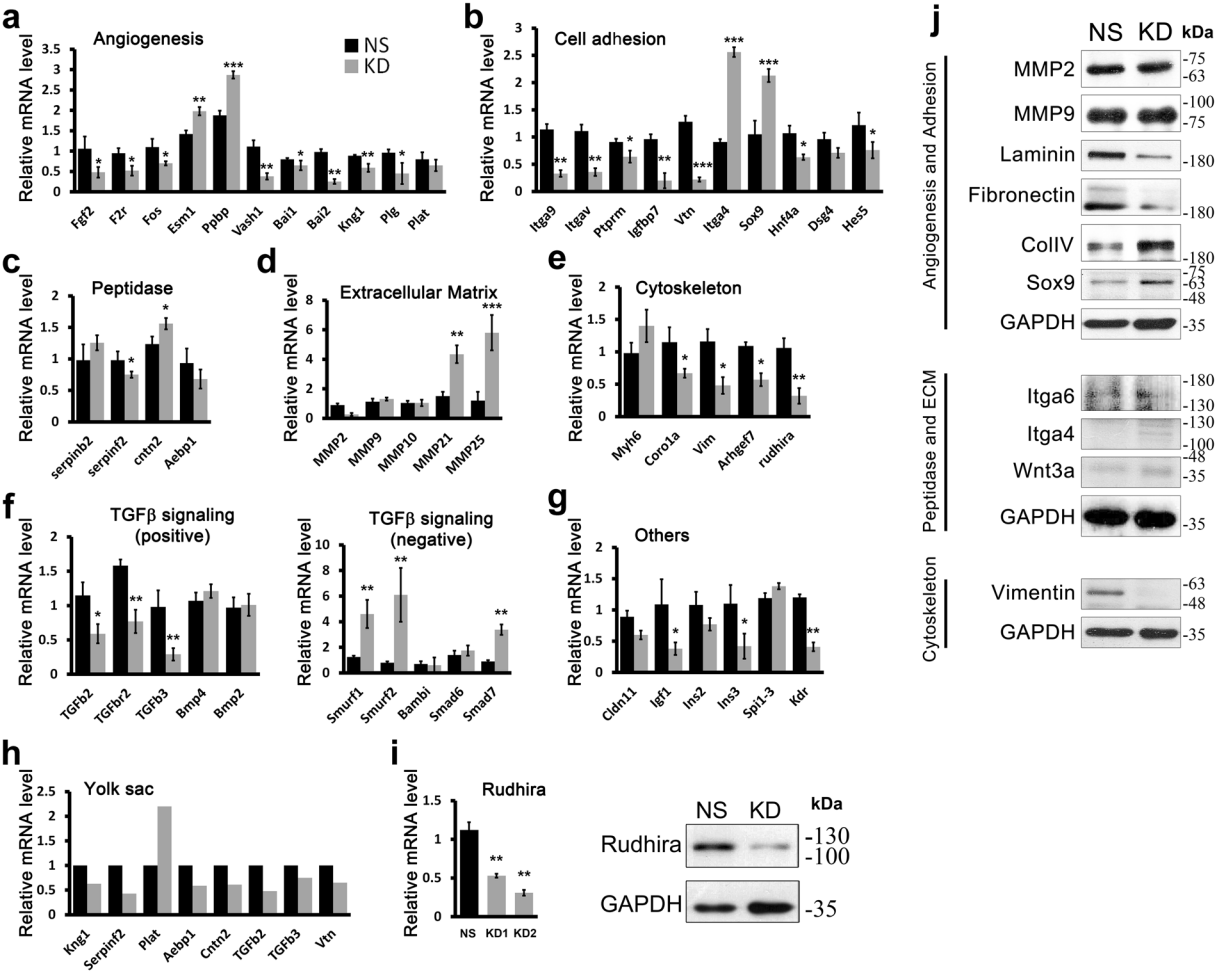
Rudhira depletion deregulates multiple networks essential for angiogenesis. (**a**–**f**) qRT-PCR on non-silencing (NS) and *rudhira* shRNA (KD) SVEC cell lines of selected differentially expressed genes and biological processes significantly deregulated in *rudh*^−/−^ yolk sac compared to control (see Fig. S3). (**g**) Additional genes relevant to vascular development were also validated by qRT-PCR on KD SVEC lines. (**h**) Confirmation of array results by qRT-PCR on control (WT) (black bar) and *rudh*^−/−^ (grey bar) yolk sacs. (**i**) Validation of *rudhira* knockdown endothelial cell line (SVEC) was confirmed by qRT-PCR and immunoblot. (**j**) Western blotting validation of protein level changes in representative candidate molecules from deregulated functional categories. Error bars indicate standard error of mean (SEM). Results shown (**a**–**j**) are a representative of at least three independent experiments with at least three biological replicates taken into account. Statistical analysis was carried out using one-way ANOVA. *p < 0.05, **p < 0.01, ***p < 0.001.

To identify the processes affected by depletion of Rudhira leading to its cardiovascular phenotypes, we performed whole transcriptome-based analysis of gene expression in *rudhira* knockout yolk sac and embryos at E9.5 (Fig. 3). Since Rudhira localization is dynamic and like other cytoskeletal elements, would likely change with tissue manipulation such as isolation and culture of cells, we chose to analyze intact tissue to understand how Rudhira affects the transcriptome. While embryos at E9.5 have a diverse set of derivatives of all three germ layers, yolk sacs are primarily made of primitive endoderm and mesoderm, the latter comprising mainly endothelial and hematopoietic lineages. Hence, we separately analyzed *rudh*^*−/−*^ embryos and yolk sacs. Since the role of Rudhira in endothelial cell migration and vessel formation *in vitro* is well established^8^ and also because knockout embryos have vessel patterning defects, we extensively validated the transcriptome data by quantitative PCR-based expression analysis of yolk sac and endothelial cell line (Fig. 4).

Volcano plot based method was used to visualize the transcripts that are two-fold differentially expressed in yolk sac (Fig. 3a). 3291 unique probes showed 2-fold or greater statistically significant changes in gene expression (Table S2). Of these 546 were downregulated and 334 upregulated in embryo and 566 downregulated and 1960 upregulated in yolk sac (Fig. 3b). 29 downregulated and 43 upregulated genes were common between embryo and yolk sac (Fig. 3b and Table S3). Unsupervised hierarchical cluster analysis showed that genes with similar expression patterns were clustered together with branch distance proportional to their similarity in expression pattern. A distinct subset showed reciprocal expression between embryo and yolk sac (Fig. 3c). Interestingly the majority of clustered genes were mainly upregulated in the yolk sac (Fig. 3c,d), while the embryo had a more balanced distribution in each cluster (Fig. 3c). To define how changes in gene expression caused by *rudhira* depletion may influence vascular development and remodeling, we functionally annotated the data using DAVID (**D**atabase for **A**nnotation, **V**isualization and **I**ntegrated **D**iscovery) and found that genes linked to many biological pathways were enriched. Key deregulated biological categories were identified (Fig. 3e, Table S4).

Analysis of the entire data set showed greater variation between duplicates of embryo than yolk sac, possibly because of higher heterogeneity in the embryonic tissue. Hence for further analysis we focused on yolk sac data as it is also one of the primary sites of vascular remodeling and shows early Rudhira expression. Significant expression changes were seen in genes that relate to a range of processes or pathways, which could impact on vascular development and remodeling (Figs 3e, 4 and Table S5). Gene ontology analysis of the common genes identified principal biological processes affected by the loss of *rudhira* with a Z score above 2.5. Important regulators of cellular processes such as angiogenesis/blood vessel remodeling, extracellular matrix, regulation of proteolysis, negative regulation of peptidase activity and cell projection organization were identified. Further, key molecular families involved in cytoskeletal remodeling, cell adhesion, cell migration and TGFβ and VEGF pathways, which are all important during angiogenesis, were connected by Rudhira/BCAS3 allowing us to identify the Rudhira network in angiogenesis (Fig. 3f and Table S6).

### Identification of regulatory networks and nodes regulated by Rudhira

A total of 140 genes from cluster analysis were enriched in the key gene ontology (GO) and pathways identified (Table S5) with a significance criterion of p < 0.05. Further, we were able to associate GOs and pathways known to co-operate during vascular development and remodeling namely adhesion, angiogenesis, cytoskeleton, ECM organization, peptidase activity and TGFβ signaling (Fig. 3e). Genes differentially expressed in these six processes were subjected to unsupervised hierarchical clustering to identify molecular signatures (Fig. S3a–f). An interaction network of significant GO terms was assembled into a GO map to depict the relationship among prominent functional categories (Fig. S3a–f). Subsequent verification of expression data was carried out for key genes known to mediate these processes (Fig. 4a–j) by transcript (Fig. 4a–h) or protein (Fig. 4i–j) analysis. 51 out of the 3407 genes that showed significant variation from control in the knockout yolk sac were validated by qRT-PCR on cDNA generated from *rudhira* knockdown and non-silencing control endothelial cell line RNA (Fig. 4i) and 70% of these (36/51) agreed with the high-throughput array data (Fig. S3a–f and Fig. 4a–g). Changes in expression level of selected candidates were further validated using cDNA generated from fresh E9.5 yolk sac RNA (Fig. 4h). The protein levels of representative molecules important for angiogenesis (Sox9, Wnt3a), adhesion (Itga4, Itga6), ECM (Fibronectin, Laminin and Collagen, MMP2, MMP9) and cytoskeletal organization (Vimentin) were tested by western blotting (Fig. 4j and Fig. S5). We find that in all cases the protein expression data corroborates that seen by microarray or RT-PCR and is in agreement with the phenotype observed. However, as the net effect on function cannot be assessed from a few protein levels, we also carried out functional assays on *rudhira* depleted cells.

### Endothelial deletion of *rudhira* leads to cardiovascular and extraembryonic vasculature defects

**Figure 5:**
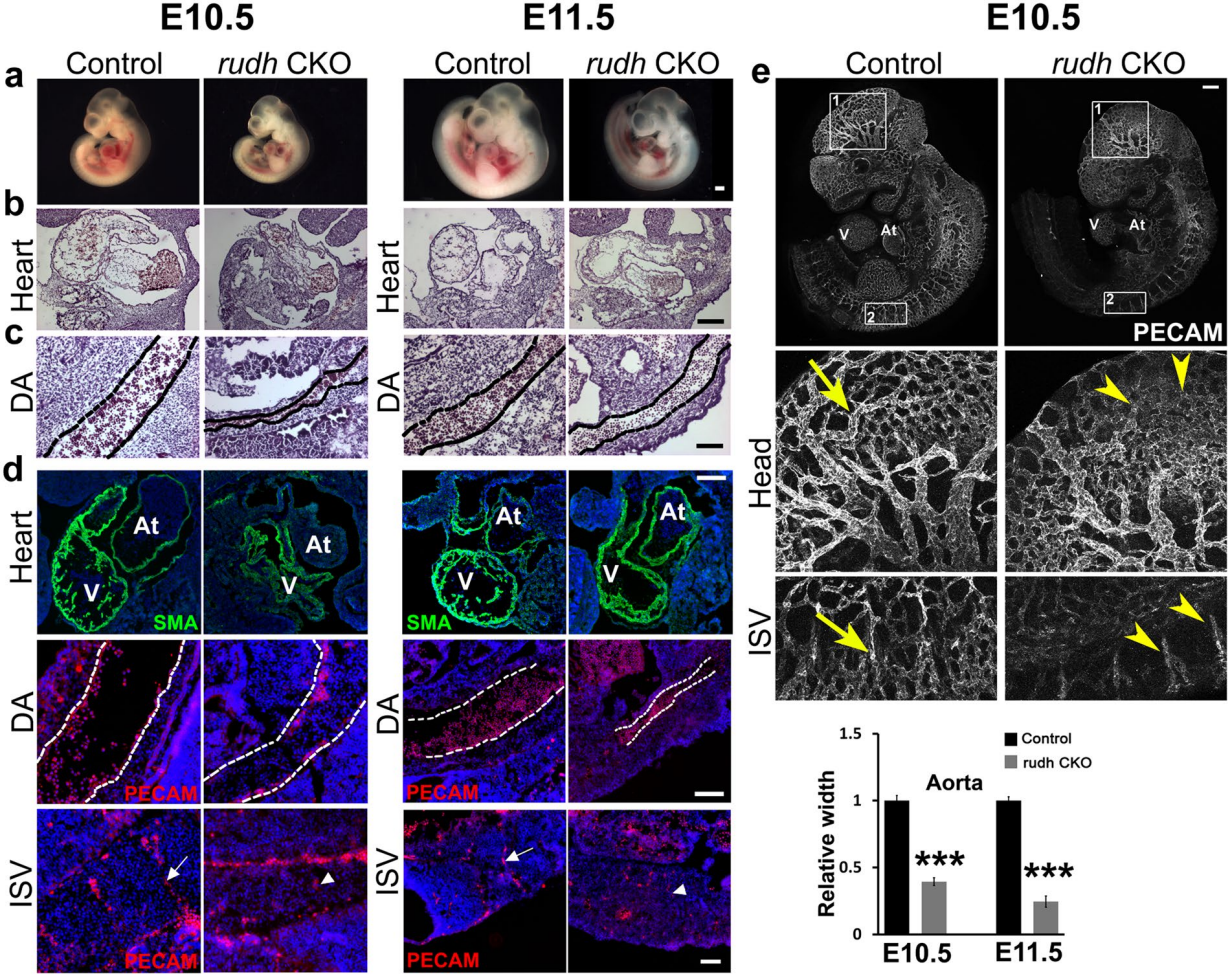
Endothelial Rudhira is essential for vascular patterning. Control and *rudh*^CKO^ at E10.5 and E11.5 were analyzed as indicated. (**a**) Unstained embryos, (**b**,**c**) Histological analysis showing comparison of heart and dorsal aorta (DA). (**d**) Immunostaining analysis of heart, dorsal aorta (DA) and intersomitic vessels (ISVs) using myocardial marker SMA, primitive erythroid marker Ly76 and vascular marker PECAM. Nuclei are marked by DAPI (Blue). Black or white dotted lines mark the boundary of DA. Graph shows quantitation of dorsal aorta width in the thoracic region. (**e**) Whole mount PECAM staining in control and *rudh*^CKO^ embryos as indicated, with magnified view of head and intersomitic vessels. Arrows: normal vascular patterning; arrowheads: irregular and discontinuous vasculature. N = 5 embryos. Results shown are a representative of at least three independent experiments with at least three biological replicates. Statistical analysis was carried out using one-way ANOVA. Scale bar: (**a**) 500 μm; (**b**,**c**) 100 μm; (**d**) E10.5 and E11.5 heart: 200 μm, E10.5 and E11.5 DA: 50 μm, ISV: 50 μm; (**e**) 500 μm. *p < 0.05, **p < 0.01, ***p < 0.001. The observed chi-square values for two degrees of freedom were 0.88 (E9.5), 2.62 (E11.5), 2.62 (E12.5), 25.68 (E13.5) and 37.5 (Postnatal).

**Figure 6:**
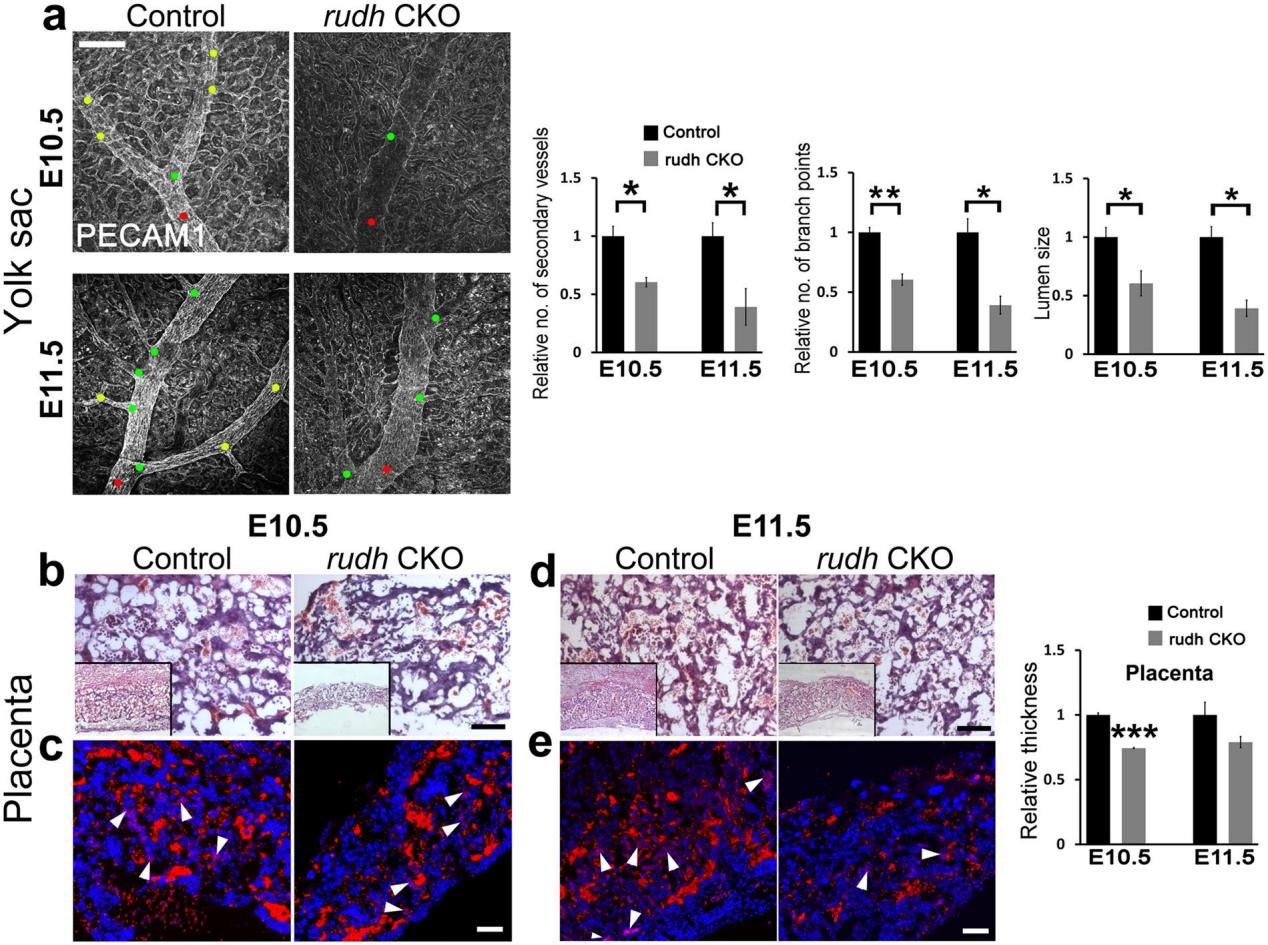
Endothelial deletion of *rudhira* leads to unpatterned extraembryonic vasculature. (**a**) Yolk sac vasculature marked by PECAM staining in control and *rudh*^CKO^ (*rudhfl/fl;TekCre+*) at E10.5 and E11.5. Red dot: primary blood vessel; green dot: secondary blood vessel; yellow dot: tertiary blood vessel. Graphs show quantitation of number of secondary vessels, branch points and lumen size in control and *rudh*^CKO^ at E10.5 and E11.5. (**b**–**e**) Placental morphology seen by histological analysis (b, d) or immunofluorescence staining (**c**,**e**) in sections of control and *rudh*^CKO^ as indicated. Boxed areas show lower magnification of placentas (**b**,**d**). Immunostaining shows fetal and maternal blood cell pockets marked by Ly76. Nuclei are marked by DAPI (Blue). Arrows: maternal blood cells; arrowheads: fetal blood cells. de: decidua, sp: spongiotrophoblast, lb: labyrinth, ch: chorion. Graph shows quantitation of placental thickness. Results shown are a representative of at least three independent experiments with at least three biological replicates. Statistical analysis was carried out using one-way ANOVA. Scale bar: (**a**) 200 μm; (**b–e**) 100 µm. *p < 0.05, **p < 0.01, ***p < 0.001.

Since Rudhira is expressed primarily in early embryonic vascular development and neo-angiogenesis and *rudhira* null embryos have vascular defects, we crossed *rudhira* floxed mice with *Tie-2-Cre* for tissue-specific ablation (*rudhira*^*flox/flox*^; *TekCre*^+^ abbreviated to *rudh*^CKO^) of the *rudhira* locus. *Tie2Cre/TekCre* has been used to delete genes in endothelial and hematopoietic lineages. Since Rudhira is primarily expressed in endothelial cells, we utilized *TekCre* to study the function of endothelial Rudhira. However, the contribution of Rudhira expressed in the hematopoietic compartment cannot be ignored. Similar to ubiquitous knockout, *TekCre*-mediated deletion of *rudhira* was validated at RNA and protein levels, and gave no live homozygous pups (Table S1b, Fig. S4a–c). Dramatic reduction in the RNA and protein levels of Rudhira upon *TekCre* mediated knockout confirmed that at this stage Rudhira is primarily expressed in the endothelial cells. Histological analysis as well as immunostaining for cardiovascular markers showed that like *rudh*^*−/−*^, *rudh*^*CKO*^ embryos had collapsed, smaller heart chambers and reduced endocardium development. Also, the major blood vessels including dorsal aorta and intersomitic vessels were discontinuous and improperly patterned (Fig. 5a–d). Whole mount immunostaining of *rudh*^CKO^ embryos with anti-PECAM1 antibodies showed striking defects in the morphology and vasculature of the head and intersomitic vessels (ISVs) in all mutant embryos analyzed at E10.5 (Fig. 5e). Control embryos had a well formed vascular network comprising major vessels giving rise to intricate secondary and tertiary branches (Fig. 5e, arrows). Although less severe, *rudh*^CKO^ embryos showed disorganized head vasculature with defective vessel sprouting, reduced capillaries and impaired branching of intersomitic vessels (ISVs) that failed to sprout into fine capillaries (Fig. 5e arrowheads). *Rudh*^CKO^ yolk sacs had reduced branching from major vessels, vessel fusion and loss of branch hierarchy at both E10.5 and E11.5 (Fig. 6a). Further, placental thickness was reduced at both E10.5 and E11.5 (Fig. 6b–e). Fetal vessel invasion was comparable to control at E10.5 (Fig. 6b,c) but reduced at E11.5 (Fig. 6d,e) although not as severely as in the global knock-out. This could explain the absence of significant growth retardation in conditional mutant embryos. Thus *TekCre*-mediated deletion of *rudhira* resulted in similar phenotypes as in the global knock-out. These results indicate that endothelial *rudhira* is essential for remodeling the vascular network.

### Rudhira is essential for endothelial sprouting angiogenesis

**Figure 7:**
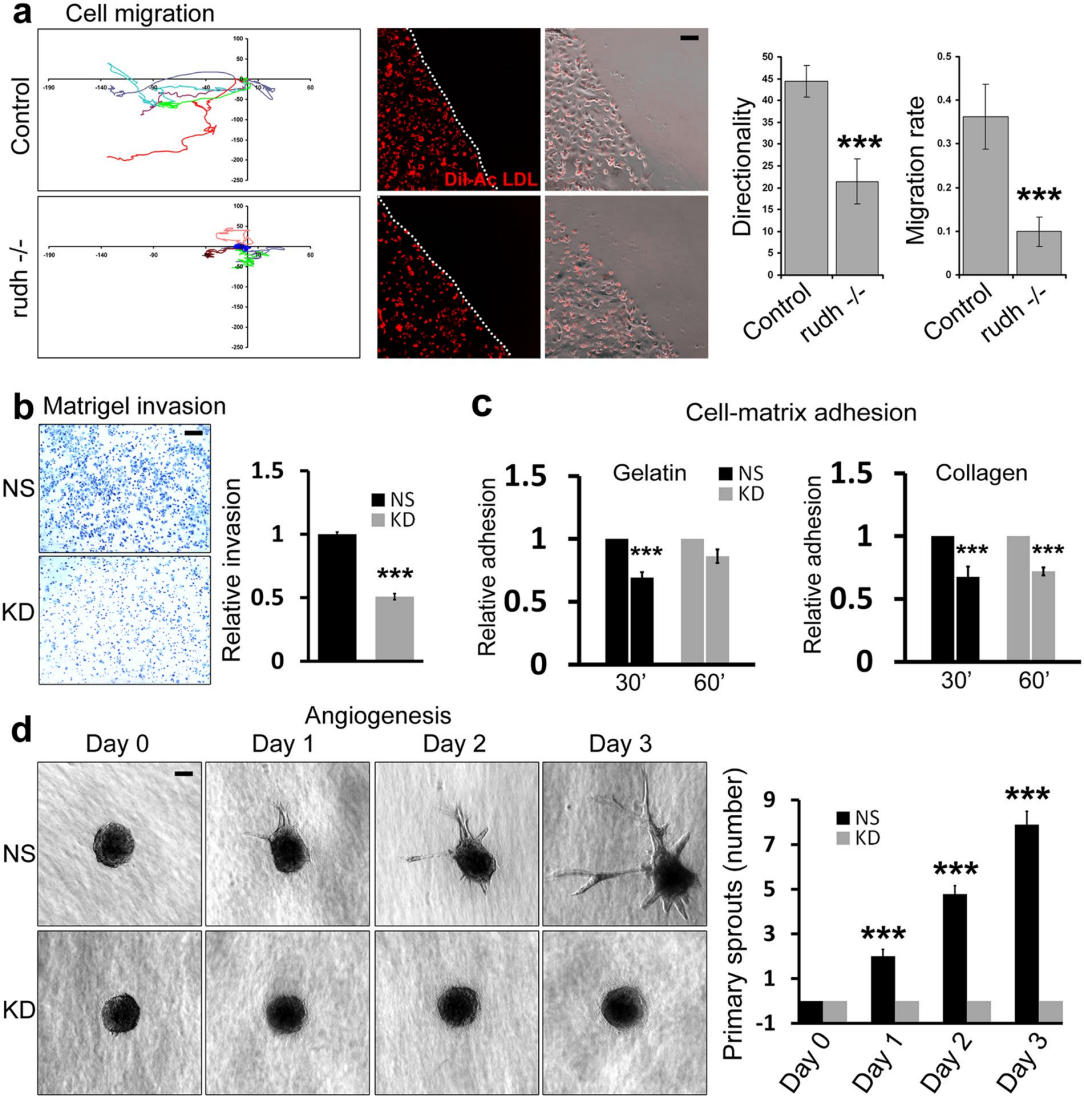
Rudhira is essential for sprouting angiogenesis. (**a**) Migration tracks of control and *rudh*^−/−^ yolk sac endothelial cells subjected to wounding assay. Quantification showing the rate of migration and directionality compared between control and *rudh*^−/−^ yolk sac endothelial cells (marked by DiI-Acetylated LDL). Dotted line indicates the wound margin. Error bar indicates mean ± SD of a total of 14 cells. (**b**) NS and KD SVEC cells were tested for invasion using a Matrigel transwell-invasion assay. Graph indicates the relative invasion of cells 24 h post seeding as measured by Crystal Violet absorbance at 590 nm (**c**) NS or KD ECs were compared for adherence when plated on gelatin or collagen. Graphs indicate the relative adhesion of cells in 30 and 60 min post seeding as measured by Crystal Violet absorbance at 550 nm. (**d**) Collagen-based spheroid sprouting assay with spheroids formed from non-silencing (NS) and *rudhira* shRNA (KD) SVEC lines. Graph shows quantitation of the number of primary sprouts over time in days. Error bars indicate standard error of mean (SEM). Results shown are a representative of at least three independent experiments with at least three biological replicates. Statistical analysis was carried out using one-way ANOVA. Scale bar: (**a**,**b,d**) 100 μm. *p < 0.05, **p < 0.01, ***p < 0.001.

The embryonic lethality and reduced vascular patterning in *rudhira* null embryos could be due to a ubiquitous role for Rudhira and/or a specific requirement in endothelial cells. In both null and CKO mutants ECs are specified but vessel patterning is aberrant, indicating the process depends on endothelial Rudhira. Vascular pattern and remodeling requires proper endothelial cell migration and vessel sprouting. We showed earlier that Rudhira is essential for EC migration^8^. As expected and in concordance to the previous cell line data, the cells derived from *rudhira* knockout yolk sacs also showed retarded migration rates and a loss of directionality (Fig. 7a). Transcriptome analysis revealed that Rudhira also affects adhesion and extracellular matrix remodeling, which are key processes in sprouting angiogenesis. Angiogenesis requires localized remodeling of extracellular matrix (ECM) and cell-cell and cell-matrix adhesions, leading to cell invasion into the ECM and extension of the vessel sprouts. In a Matrigel invasion assay *rudhira* KD ECs showed reduced invasion (Fig. 7b). Also cell adhesion was reduced on gelatin and collagen matrices upon *rudhira* depletion (Fig. 7c). Finally, we tested *rudhira* knockdown ECs in an *in vitro* spheroid sprouting angiogenesis assay. While control cells expressing non-silencing shRNA (NS) showed primary, secondary and tertiary sprouts that increased in length over the time course of the assay, *rudhira* KD ECs failed to sprout (Fig. 7d). Thus Rudhira is essential for sprouting angiogenesis, indicating that the vascular defects in the mouse knockout are primarily due to lack of endothelial Rudhira and not mere placental insufficiency or general developmental defects.

## Discussion

Vertebrate blood vessel formation involves *de novo* differentiation of endothelial cells (ECs) to form a primary plexus, which is pruned and patterned into a hierarchical network by angiogenesis^14^. A fine balance of pro- and anti-angiogenic cues maintains ECs in a quiescent state^15,16^. Perturbation of this balance leads to endothelial activation and cytoskeletal changes resulting in sprouting, migration and maturation^17,18^. EC migration and tube formation are key steps during angiogenesis, however, molecular mechanisms that operate are incompletely defined. Random and retarded EC migration results in an unpatterned and often leaky vasculature^19,20^.

We report here that global or endothelial deletion of a cytoskeletal protein, Rudhira, resulted in mid-gestation lethality with severe defects in cardiovascular patterning. Based on a transcriptome analysis we identified key steps in blood vessel remodeling such as cell adhesion, migration, extracellular matrix components and TGFβ signaling that are regulated by the action of Rudhira. We describe, for the first time, a regulatory network mediated by Rudhira with reference to its interacting partners at both binary (regulatory) as well as physical levels (Fig. 3f).

From E8.5 in the mouse yolk sac, blood flow dictates vessel fusion and directional cell migration resulting in vascular remodeling^21^. Cardiac remodeling defects seen in *rudhira* mutants may also impair circulation and contribute to the vascular remodeling abnormalities. Hence all abnormalities noted in *rudhira* null embryos could be due to placental insufficiency and generalized growth retardation. However, our analysis of the CKO mutants shows conclusively that there is an essential role for endothelial Rudhira in vascular patterning as well as survival.

Loss of Rudhira affects expression of the endothelial cell transcriptome. Genes implicated in multiple processes important for angiogenesis such as cell adhesion, invasion, matrix organization and degradation are deregulated in the *rudhira* knockout. It is likely that imbalance in action of these processes and aberrant signaling result in grossly defective angiogenesis in *rudhira* knockout. Further, the TGFβ pathway, which functions in a multitude of angiogenic processes was over-represented in the *rudhira* knockout, suggesting a role in TGFβ signalling, though other signaling pathways cannot be ruled out. Our analysis provides tools to investigate further the precise roles of Rudhira in the TGFβ pathway and in sprouting angiogenesis and vascular remodeling.

Given the large number of signals and their varying levels that cells encounter, it is unlikely that they respond only to one or the same concentration all across the organism. A robust response over a range of signals is likely mediated by molecules that can crosstalk with a wide variety of processes. The cytoskeleton is ideally positioned for this role. Endothelial cells respond to a variety of signals resulting in a limited repertoire of cytoskeletal changes, which in turn determine cell phenotype. The presence or absence of cell type-specific components such as Rudhira could provide decisive control of endothelial cell behavior in response to a varying milieu of signals. For example, Rudhira could have distinct context-dependent tissue-specific roles in regulating the cytoskeleton in EC migration as reported earlier^8^ or the transcriptome in development as reported here. As both angiogenic remodeling and sprouting angiogenesis occur in embryogenesis, our study opens up new avenues for understanding vascular patterning in development and disease.

## Materials and Methods

### Generation and validation of *rudhira* knockout mice

The *rudhira* locus (Chromosome location: 11:85166669-85639560:1) was targeted at exon 6 by homologous recombination using a targeting vector (Fig. 1a) in TT2 mouse embryonic stem cells in order to generate a floxed *rudhira* allele^22^. Recombinants were selected by G418 resistance and genomic analyses with Southern hybridization, and microinjected into ICR (CD-1) 8-cell stage embryos. Resulting chimeras were then crossed to C57BL/6N to obtain germ line transmitted recombinant progeny. The heterozygous recombinant alleles (*rudh*^*flox/*+^) were then confirmed by Southern blotting (Supplementary Fig. 1a). Matings between heterozygous *rudh*^*flox/+*^ mice were set up to obtain homozygous floxed *rudhira* mice. Deletion of *rudhira* ubiquitously using *CMV-Cre*^23^ or tissue- specifically by crossing to the endothelial specific *Tie-2-Cre*^24^ gave heterozygous knockout mice which were phenotypically normal with viability and fecundity similar to control littermates. Heterozygotes (*rudh*^*fl/+*^; *Cre*^*+*^) were crossed to *rudhira* floxed mice (*rudh*^*fl/fl*^) and the progeny analyzed as described in Results. All animal experimental protocols were approved by Institutional Animal Ethics Committee (IAEC) of JNCASR (Project number MSI006) and the Institutional Animal Ethics Committee (IAEC) of NCBS (Project number KVR-2(2)/2015). All animals were maintained and experiments performed according to the guidelines of the animal ethics committees of JNCASR, NCBS and RIKEN, CDB. *Rudhira* floxed mice (Accession No. CDB0664K: http://www2.clst.riken.jp/arg/mutant%20mice%20list.html) and knockout mice were validated by genotyping (see Fig. 1 and Fig. S1) (For genotyping, see the supplementary Materials and methods.).

### Generation of knockdown EC lines

*Rudhira*/*BCAS3*shRNA vectors (715, 716), and scrambled (non- silencing) control vector (TR30015) (Origene, USA) were microporated into SVEC (mouse endothelial cell line) and selected for stable line generation. (For further details, see the supplementary Materials and methods.) Control cell line is referred to as ‘NS’ and *rudhira* knockdown as ‘KD’.

### Quantitative RT-PCR (qRT-PCR)

Gene expression changes were quantitated by real-time PCR on cDNA generated from control or mutant RNA. (For further details, see the supplementary Materials and methods.). Primers used are provided in Supplementary Table S7.

### Immunostaining and Immunohistochemistry

Yolk sac or embryos dissected at desired stages between E7.5 to E11.5, were fixed in 4% paraformaldehyde and processed for cryosectioning (embryos) and immunostaining using standard procedures^25^. Samples were viewed and imaged using bright field, phase contrast or fluorescence microscopy (For further details, see the supplementary Materials and methods.)

### Western blot analysis

25 μg lysate from control or *rudhira* knockdown cell lines of SVEC was used for western blot analysis by standard protocols. Blots were cut into strips and incubated with primary antibodies as indicated: MMP2, MMP9 (Cell Signaling Technology), Fibronectin, Vimentin, GAPDH (Sigma Chemical Co., USA), Sox 9 (RND Systems), Laminin, ColIV, Wnt3a (Abcam), Itga6, Itga4 (BD Biosciences) and BCAS3 (Bethyl Labs, USA). HRP conjugated secondary antibodies against appropriate species were used and signal developed by using Clarity Western ECL substrate (Biorad, USA).

### Cell migration assay

E9.5 yolk sacs were washed in phosphate buffered saline (PBS), minced and dissociated in 0.2% collagenase type IV (GIBCO/BRL) at 37 °C for 5 minutes, washed, pelleted and resuspended in culture medium (DMEM, 20% fetal calf serum, 1 × Glutamax, 1 × antibiotics and 50 μg/ml Endothelial Cell Growth Supplement (ECGS) (Sigma Chemical Co., USA)) and plated onto 0.1% gelatin coated dishes. Confluent monolayers were incubated with 5 μg/ml DiI-Ac-LDL (Invitrogen) for 4 hours to mark endothelial cells, then scratched and monitored for cell migration in real time as described before^8^. Rate of wound closure was calculated as the distance covered per min for around 200 cells at each wound margin. At least 30 cells per margin were selected randomly and monitored in real time post-wounding for the tracking assay and analyzed for their directionality.

### Cell-matrix adhesion assay

Control (NS) or *rudhira* knockdown (KD) cells were plated at 2 × 10^4^/well onto 96 well plates that were pre-coated with collagen (20 μg/ml) or 0.1% gelatin and blocked with 0.5% BSA. After incubation of plated cells at 37 °C for the desired time, wells were washed in PBS, fixed with 4% paraformaldehyde, stained with 5 mg/ml crystal violet, solubilised with 100 μl 2% SDS solution and absorbance measured at 550 nm.

### Matrigel invasion assay

The assays and quantitation were carried out as described before^8^. Briefly, control (NS) or *rudhira* knockdown (KD) cells were serum-starved for 12 h and 20000 cells were plated onto the upper chamber of transwell filter inserts with 8 μm pore size, 24-well format (Costar), pre-incubated with 0.03 mg/ml Matrigel for 4 h to initiate the assay. After the assay, the dye was extracted in methanol and absorbance measured spectrophotometrically at 590 nm.

### Spheroid sprouting assay

Endothelial spheroids were prepared as described^26^ with few modifications. 750 cells each of NS or KD lines were taken for spheroid formation in a round-bottom non-adherent 96-well dish (Costar), in 1% CMC (carboxy methyl cellulose) in 10% FBS in DMEM. The spheroids formed were transferred to collagen gels (Rat tail, Type I, Invitrogen) with a final concentration of 2.5 mg/ml. Gels were overlaid with 200 µl of 10% FBS in DMEM and the sprouting was monitored for 4–5 days.

### Microarray sample processing and data analysis

E9.5 embryos of control and knockout littermates generated by crossing to *CMV-Cre*, were identified by embryonic tail genotyping. Embryo and yolk sac were separated and total RNA extracted from each by TRIzol method. (For further details, see the supplementary Materials and methods.).

All reported data are MIAME compliant and raw data have been deposited in NCBI’s Gene Expression Omnibusand are accessible through GEO Series accession number (http://www.ncbi.nlm.nih.gov/geo/query/acc.cgi?acc=GSE69204) a MIAME compliant database^27^. All other data generated or analyzed during this study are included in this published article (and its Supplementary Information files).

### Quantification and Statistical analyses

Statistical significance analyses were performed using One Way ANOVA in the Data Analysis package in Microsoft Excel. p < 0.05 was considered significant. Statistical significance for frequency of embryos per genotype was calculated using chi-square test.

## Electronic supplementary material

Supplementary information

S2

S3

S4

S5

S6
