# Supplementary material for: Rudhira/BCAS3 is essential for mouse development and cardiovascular patterning
Source: Sci Rep. 2018 Apr 4;8:5632. doi: 10.1038/s41598-018-24014-w (PMC5884795; doi:10.1038/s41598-018-24014-w)
Supplement: Supplementary file 1 — Supplementary information [file 41598_2018_24014_MOESM1_ESM.pdf]

Rudhira/BCAS3 is essential for mouse development and cardiovascular patterning.

Ronak Shetty[1#], Divyesh Joshi[1#], Mamta Jain[1], Madavan Vasudevan[3], Jasper Chrysolite Paul[1] , Ganesh Bhat[1], Poulomi Banerjee[1], Takaya Abe[2], Hiroshi Kiyonari[2], K. VijayRaghavan[4] and Maneesha S. Inamdar[1,5*]

Inventory of Supplementary Information

1. Supplementary information
    a. Supplementary text
    b. Supplementary Figure 1
    c. Supplementary Figure 2
    d. Supplementary Figure 3
    e. Supplementary Figure 4
    f. Supplementary Figure 5
2. Supplementary Table 1
3. Supplementary Table 2
4. Supplementary Table 3
5. Supplementary Table 4
6. Supplementary Table 5
7. Supplementary Table 6
8. Supplementary Table 7
9. Revised main text with tracked changes
10. Revised supplemental text with tracked changes

Rudhira/BCAS3 is essential for mouse development and cardiovascular patterning.

Ronak Shetty[1#], Divyesh Joshi[1#], Mamta Jain[1], Madavan Vasudevan[3], Jasper Chrysolite Paul[1] , Ganesh Bhat[1], Poulomi Banerjee[1], Takaya Abe[2], Hiroshi Kiyonari[2], K. VijayRaghavan[4] and Maneesha S. Inamdar[1,5*]

Supplementary materials and methods

Genotyping

Genomic DNA was extracted from cultured ectoplacental cone at E7.5, yolk sacs at E8.5 and tails of E9.5 or older embryos. Primers used (Table S7) to identify genotypes of *rudhira* were: RudGNWT (F and R) for wild type allele; RudGNMUTF and RudGNWTR for the floxed allele; RudLoxR2 and RudGNMUTF for excision of the floxed allele. *Cre* positive mice were identified by PCR using primers CreF and CreR. Reactions were subjected to 30 cycles of denaturation at 94°C for 1 min, annealing at 56°C for 1 min and extension at 72°C for 1 min.

Quantitative RT-PCR (qRT-PCR)

RNA from E9.5 yolk sac or endothelial cells was isolated using TRIzol reagent (Invitrogen). Reverse transcription was performed using 2 µg of DNase treated RNA and Superscript II (Invitrogen, Carlsbad, CA) according to manufacturer's instructions. Quantitative RT-PCR (qRT-PCR) was carried out using EvaGreen (BIO-RAD, CA) in Biorad-CFX96 Thermal cycler (BIO-RAD, CA). Primers used are provided in Table S7.

Immunostaining and Immunohistochemistry

Primary antibodies used were against Rudhira (1:10)[1], PECAM1 (1:50, cat. no. 557355), Flk1 (1;50, cat. no. 555307), Ly76 (1:50, cat. no. 553670) (BD Biosciences), Brachyury (1:50, cat. no. sc-17743) (Santa Cruz Biotechnology), Smooth Muscle Actin (1:10000, cat. no. A2547) (Sigma Chemical Co. USA). Secondary antibodies were coupled to Alexa-Fluor 488 or Alexa-Fluor 568 or Alexa-Fluor 633 (Molecular Probes). Cryosections were stained with haematoxylin and eosin using standard protocols.

Fluorescence Microscopy and Analysis

Bright field and phase contrast microscopy was done using a stereo zoom (SZX12 Olympus) or inverted (IX70, Olympus) microscope. Confocal microscopes (LSM 510 Meta and LSM 880 with airy scan, Zeiss) and a motorized inverted microscope with fluorescence attachment (IX81, Olympus) were used for fluorescence microscopy. Images were captured using a CCD camera (CoolSNAP, Roper Scientific) or an EM CCD camera (Andor, LucaR). All images in a set were adjusted equally for brightness and contrast using Adobe Photoshop CS2 where required.

Microarray data analysis

RNA integrity and quality were analyzed by 2100 bioanalyzer (Agilent Technologies, Santa Clara, CA). 500 ng of total RNA for each sample was used to generate biotin-labelled cRNA using IlluminaTotalPrep RNA Amplification kit according to the manufacturer's protocol (Illumina Cat. No. AMIL1791) and subjected to microarray transcriptional analysis in quadruplicate. 750 ng labelled cRNA/array was hybridized to the Illumina Mouse WG6 BeadChip (45200 transcripts, version 2) according to the manufacturer's instructions. Arrays were scanned with an Illumina Bead array Reader confocal scanner (BeadStation 500GXDW) and imported into BeadStudio software for obtaining Sample Gene Profile data. Raw data obtained in .txt format were normalized using GeneSpring GX v 12.0. Intra array Quantile normalization was done for each chip / sample. Inter array normalization was done by taking median of all the samples. Volcano plot based method was used to visualize the transcripts (Fig. 3a) that are 2 fold differentially expressed (up- or down-regulated) between any two conditions (Ex: Mutant vs. Wild type) by applying Unpaired Student's t-test for p-value calculation ($p<0.05$) and Benjamini Hocheberg based FDR correction. Pairwise comparison between samples revealed a high correlation coefficient (>99% concordance). Unsupervised hierarchical clustering of differentially expressed genes was done by Pearson Uncentered algorithm with Average linkage rule to identify up- and down- regulated gene clusters. Unsupervised hierarchical clustering of samples showed replicates grouping in the same branch with very minimal distance between them indicating reproducible measurement (Fig. 3c,d). Replicate samples from the same genotype showed tight clustering. Comparison of up and down regulated genes in both Embryo and Yolk Sac was done and represented using Venn Diagram for understanding the basal differential expression profiles between two tissues (Fig. 3b).

Gene ontology and pathway analysis

Biological analysis of differentially expressed genes was done for Gene Ontology and Pathways using DAVID tool (http://david.abcc.ncifcrf.gov/). Statistically significant ontologies and pathways were filtered

based on $p<0.05$ (Obtained using Fischer Exact Test) with Benjamini Hocheberg FDR correction. Key Gene Ontology categories and Pathways that were enriched were represented as histogram (Fig. 3e).

Biological Analysis Network (BAN)

Regulatory network (nodes and edges) underlying the differential expression upon loss of *rudhira/BCAS3* gene expression was modeled by considering the key deregulated pathways and Gene Ontologies along with the differentially expressed genes harbored as an input to BridgeIsland© software (Bionivid Technology Pvt Ltd, Bangalore, India). Output of the software results in identification of key nodes and edges enriched in a gene: process manner. Further, the output was imported to Cytoscape v 8.3[2] to visualize the biological network to understand key regulatory genes and processes. Force Directed Spring Embedded algorithm was applied to model the network and visualized. Nodes were sized based on their p-Value and colored based on their fold change in mutant in comparison to wild type sample. Experimental data from pre-published studies and from this experiment was used to create the BCAS3 regulatory network using Cytoscape V 8.3 by applying Hierarchical Layout algorithm (Fig. 3f).

Data availability

All relevant data are within the paper and its Supplementary Information files. All reported microarray data are MIAME compliant and raw data have been deposited in NCBI's Gene Expression Omnibus and are accessible through GEO Series accession number (http://www.ncbi.nlm.nih.gov/geo/query/acc.cgi?acc=GSE69204) a MIAME compliant database.

Generation of knockdown EC lines

Saphenous vein endothelial cell line (SVECs) was obtained from Kaustabh Rau, National Centre for Biological Sciences, Bangalore) and maintained in culture medium (DMEM, 10% fetal bovine serum (FBS), 1X Glutamax). *Rudhira*/*BCAS3*shRNA vectors (715, 716), and scrambled (non- silencing) control vector (TR30015) (Origene, USA) were microporated into SVEC according to the manufacturer's instructions (Neon; Life Technologies). The transfected cells were selected in puromycin containing media (1 µg/ml) for 7 days and >99% RFP positive population was obtained by fluorescence associated cell sorting (FACS) using a BD Aria II (BD Pharmingen). *Rudhira*/*BCAS3* knockdown was validated by qRT PCR and Western blot analysis.

References

1 Jain, M., Bhat, G. P., Vijayraghavan, K. & Inamdar, M. S. Rudhira/BCAS3 is a cytoskeletal protein that controls Cdc42 activation and directional cell migration during angiogenesis. *Exp Cell Res* **318**, 753-767, doi:S0014-4827(12)00037-7 [pii]

10.1016/j.yexcr.2012.01.016 (2012).

2 Shannon, P. *et al.* Cytoscape: a software environment for integrated models of biomolecular interaction networks. *Genome research* **13**, 2498-2504 (2003).

Supplementary figure legends

File: Fig_S1

Supplementary Fig. S1 (related to Figs. 1 to 4, 7). Validation and analysis of *rudhira* knockout mice. (a) Southern blotting analyses to confirm heterozygous floxed *rudhira* mice. Arrows represent the shifted band due to insertion. Positive clones are marked with asterisks. (b) Embryo sections stained for Rudhira (green) showing expression in control and *rudh*$^{-/-}$ as indicated. Nuclei are marked by DAPI (Blue). (c) Validation of *rudhira* knockout by western blotting. (d, e, e') Phase contrast images of control and *rudh*$^{-/-}$ embryos at E7.5 and E8.5. Whole mount staining for Brachyury (Green) at E7.5 and Flk1 (Red) in aortic primordia at E8.5 (boxed regions) is also shown. (e') Phase contrast and immunostaining of ~8 somite *rudh*$^{-/-}$ embryo, a stage comparable to wild type. Arrows point to discontinuous Flk1 staining in aorta. (f) Representative image showing measurements made on the embryo. Graphs show the quantitation of curved length (black line), head to heart distance (blue line), length (red line) and width (green line) of control and *rudh*$^{-/-}$ embryos. Scale bar: (b) 50 µm; (d) 200 µm; (e) 300 µm.

File: Fig_S2

Supplementary Fig. S2 (related to Fig. 1). Vascular remodeling defects in *rudhira* null embryos. Whole mount PECAM staining in control and *rudh*$^{-/-}$ yolk sacs as indicated. Boxed regions are magnified in the adjacent panels. Red dots: primary blood vessel; green dots: secondary blood vessel; yellow dots: tertiary blood vessel. Scale bar: 200 µm.

File: Fig_S3

Fig. S3. (related to Figs. 3, 4). Rudhira depletion deregulates multiple networks essential for angiogenesis. (a-f) Unsupervised hierarchical clustering computed with Pearson Uncentered algorithm with average linkage rule and regulatory networks of differentially expressed genes and biological processes significantly deregulated in *rudh*$^{-/-}$ yolk sac compared to control. Network maker program (Bionivid Technology Pvt Ltd) was used to identify the nodes and edges that form the regulatory circuit and Cytoscape V 8.0 was used to visualize the network. Edge weighted spring embedded layout was used to visualize the network.

File: Fig_S4

Supplementary Fig. S4 (related to Figs. 5, 6). Validation and analysis of *rudhira* CKO mice. (a) Embryo sections stained for Rudhira (green) and Flk1 (red) showing expression in control and *rudh*^{CKO} as indicated. Nuclei are marked by DAPI (Blue). (b, c) Validation of CKO by RT (b) and immunoblot (c). Scale bar: (a) 50 µm.

Supplementary Fig. S5 (related to Fig. 4). Full-length pictures for western blots. Full-length pictures for western blots presented in Fig. 4, S1 and S4. Membranes were cut to enable probing with multiple antibodies.

Supplementary table legends

File: Table_S1

Supplementary Table 1. Analysis of embryo genotypes and lethality mapping.

File: Table_S2

Supplementary Table 2. List of differentially regulated genes in *rudh*$^{-/-}$ embryo and yolk sac.

File: Table_S3

Supplementary Table 3. List of common genes (up-regulated and down-regulated) in *rudh*$^{-/-}$ embryo and yolk sac.

File: Table_S4

Supplementary Table 4. List of dysregulated biological categories in *rudh*$^{-/-}$ embryo and yolk sac.

File: Table_S5

Supplementary Table 5. List of genes for individual network (adhesion, angiogenesis, cytoskeleton, ECM organization, peptidase activity and TGFβ signaling).

File: Table_S6

Supplementary Table 6. List of BCAS3 pathway genes in the Cytoscape model (related to Fig 3f).

File: Table_S7

Supplementary Table 7. List of primers.

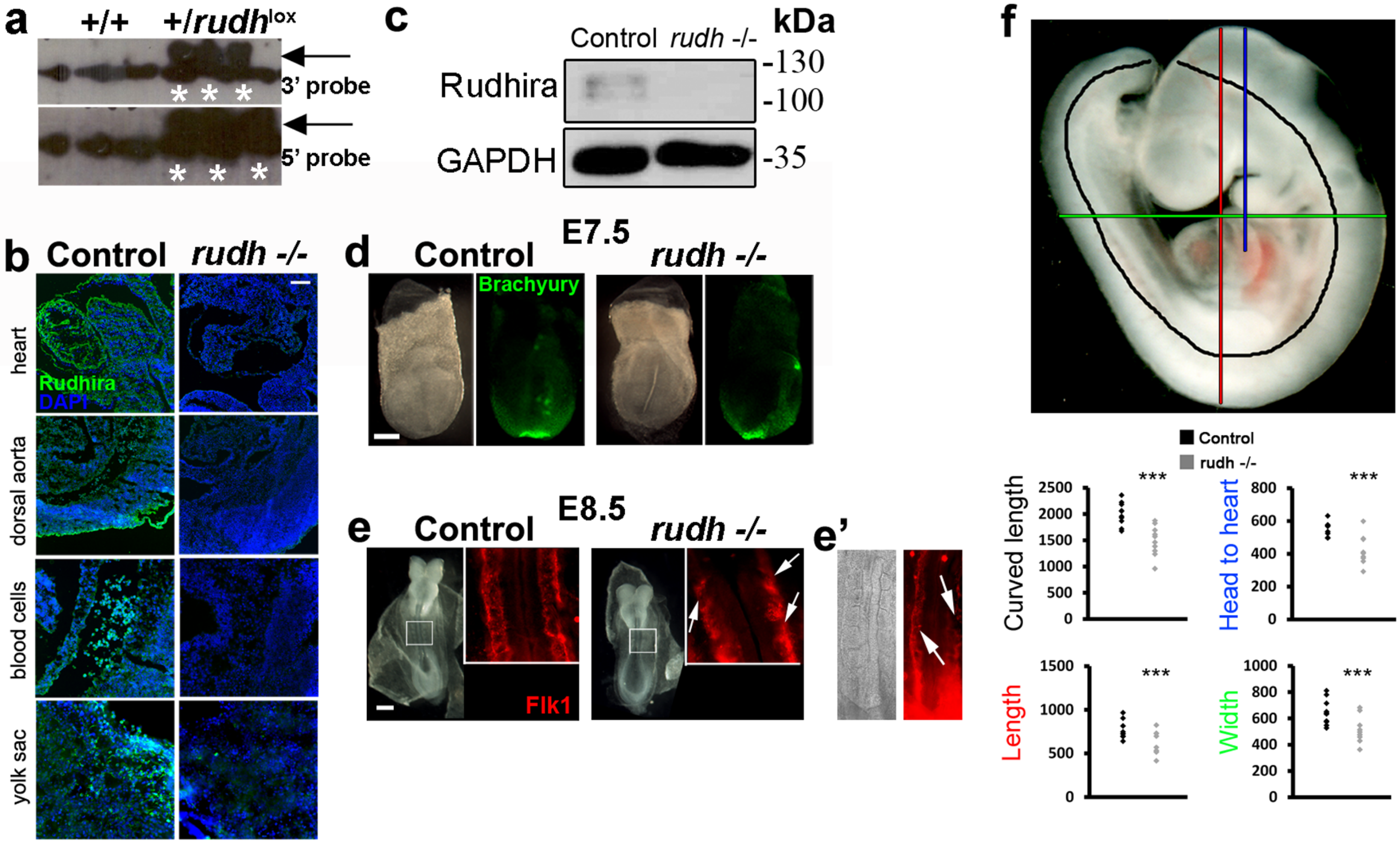

a
+/+
+/rudh lox
3' probe
5' probe
b
Control
rudh -/-
heart
Rudhira
DAPI
dorsal aorta
blood cells
yolk sac
c
Control
rudh -/-
kDa
Rudhira
GAPDH
-130
-100
-35
d
E7.5
Control
rudh -/-
Brachyury
e
E8.5
Control
rudh -/-
Flk1
e'
f
Control
rudh -/-
Curved length
Head to heart
Length
Width
***

Figure S1

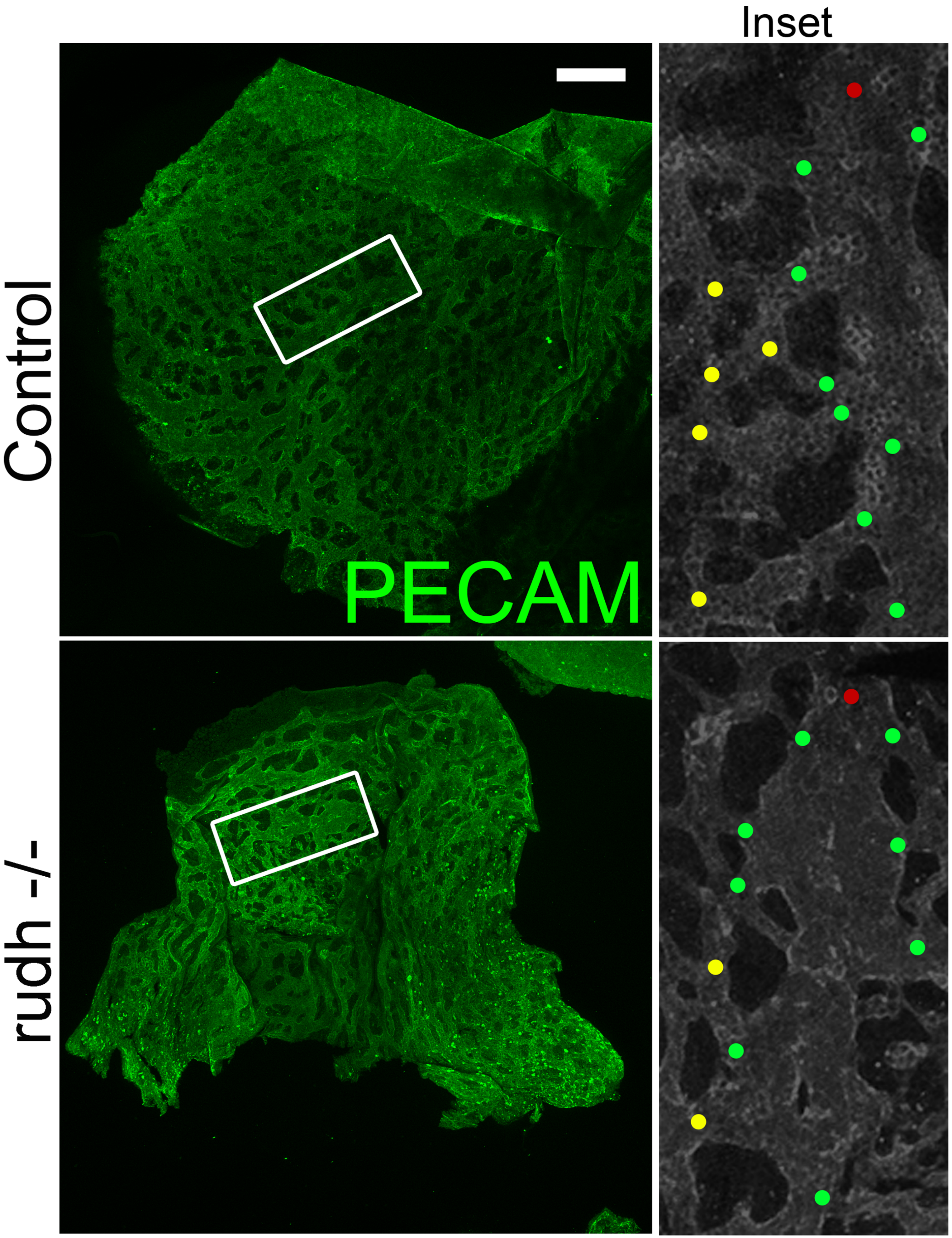

Figure S2

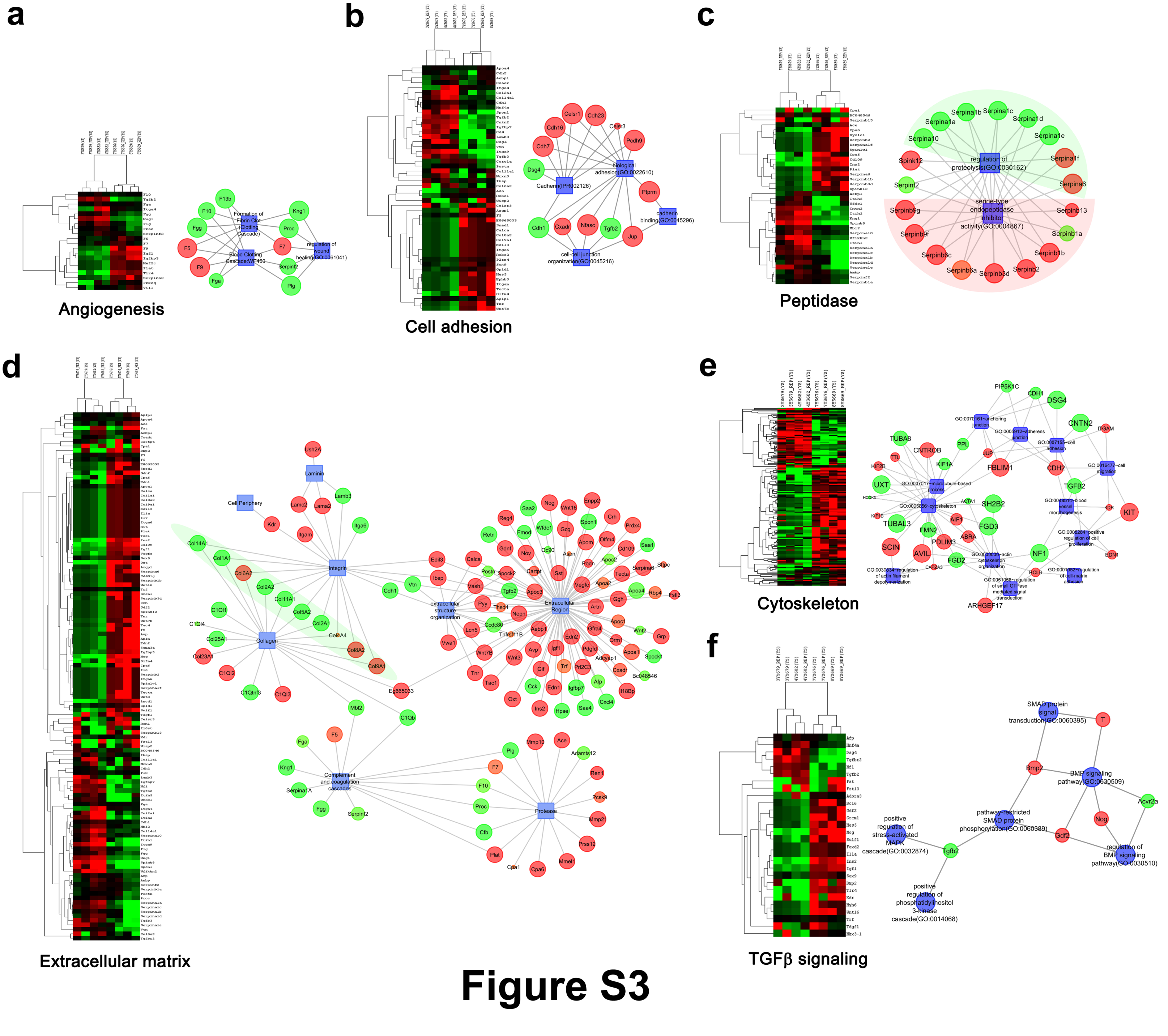
a
Angiogenesis
b
Cell adhesion
c
Peptidase
d
Extracellular matrix
e
Cytoskeleton
f
TGFβ signaling

Figure S3

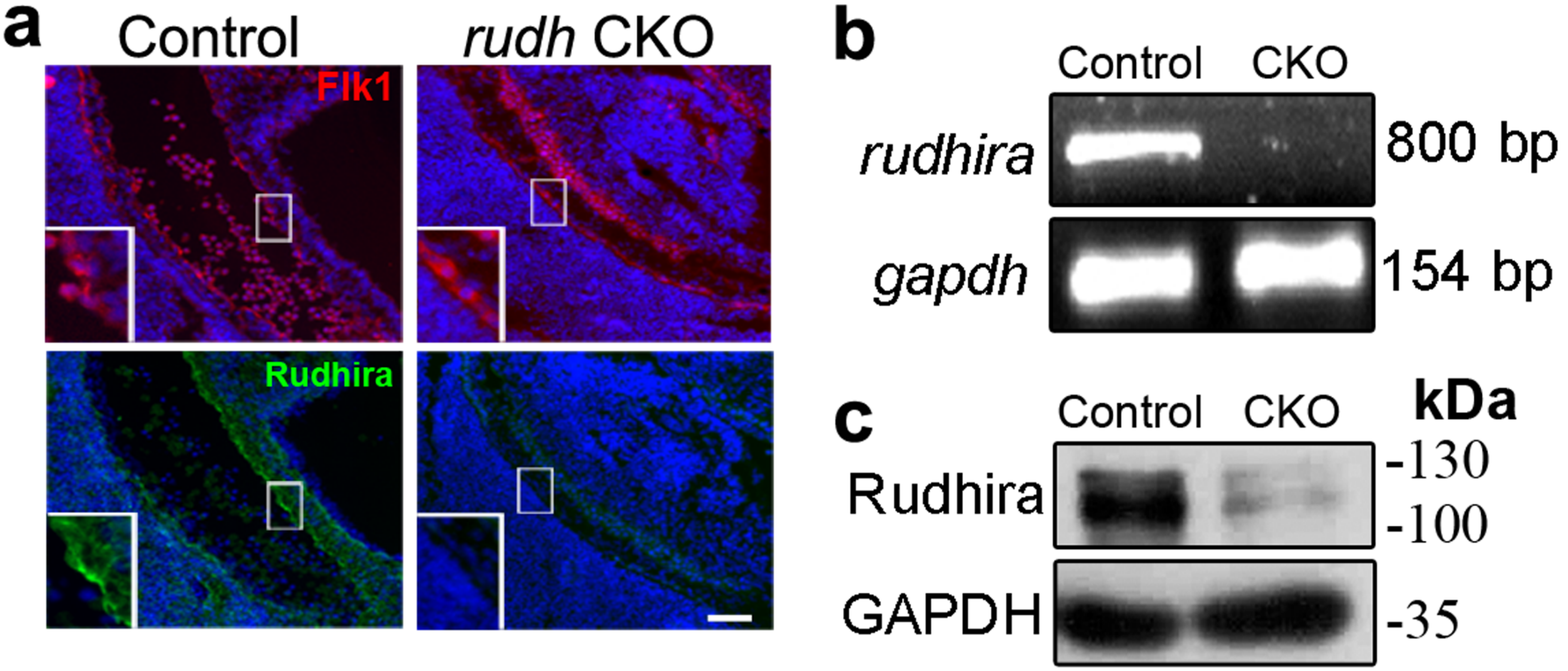
a
Control
rudh CKO
Flk1
Rudhira
b
Control
CKO
rudhira
800 bp
gapdh
154 bp
c
Control
CKO
kDa
Rudhira
-130
-100
GAPDH
-35

Figure S4

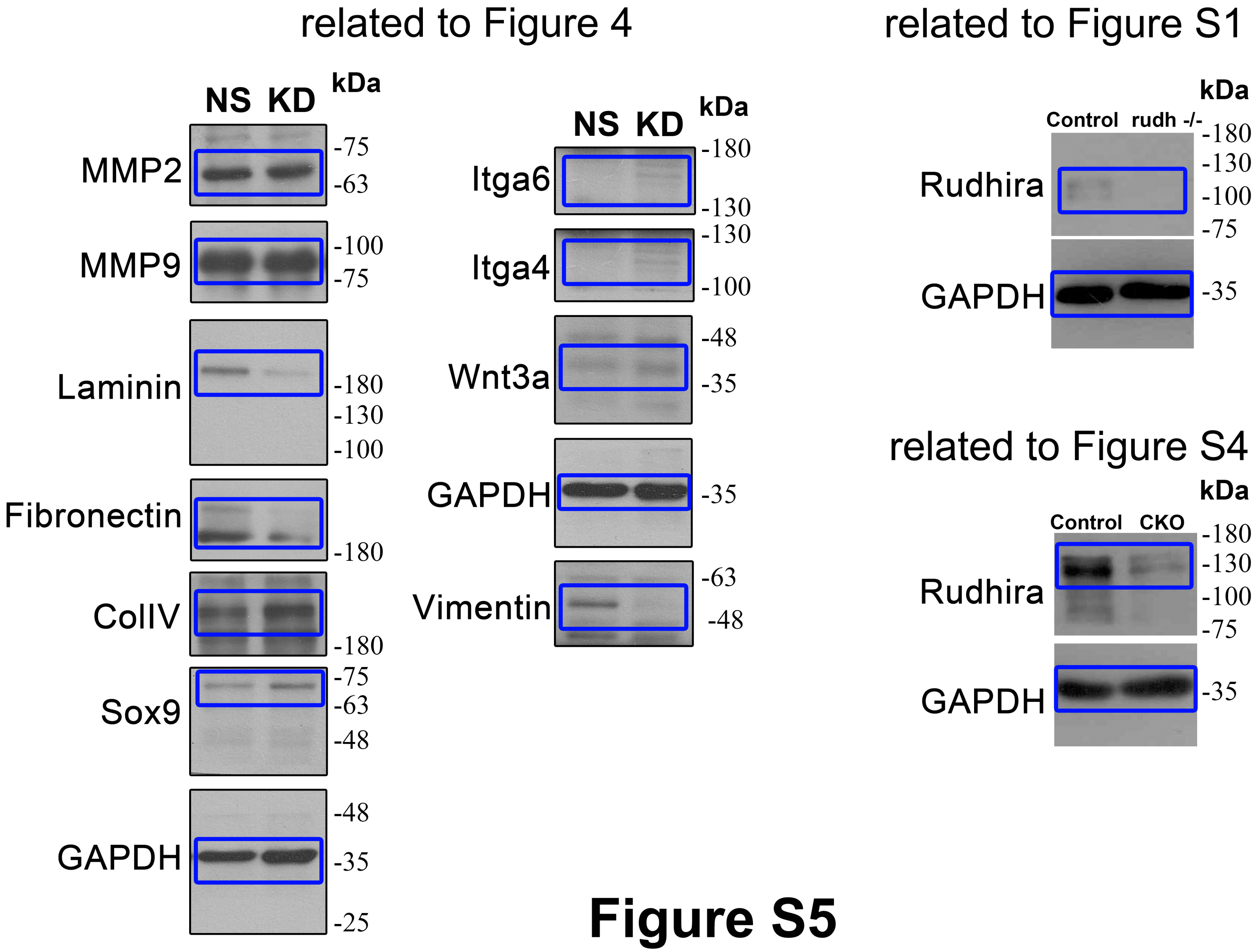

related to Figure 4
NS KD
kDa
MMP2
-75
-63
MMP9
-100
-75
Laminin
-180
-130
-100
Fibronectin
-180
ColIV
-180
Sox9
-75
-63
-48
GAPDH
-48
-35
-25
Itga6
-180
-130
Itga4
-130
-100
Wnt3a
-48
-35
GAPDH
-35
Vimentin
-63
-48
related to Figure S1
Control rudh -/-
Rudhira
-180
-130
-100
-75
GAPDH
-35
related to Figure S4
Control CKO
Rudhira
-180
-130
-100
-75
GAPDH
-35

Figure S5

A

dpc	No. of embryos	+/+	+/-	-/-
8.5	100	21 (21%)	63 (63%)	16 (16%)
9.5	235	58 (24.68%)	151 (64.25%)	26 (11.06%)
10.5	17	4 (23.53%)	12 (71%)	1 (5.88%)
11.5	13	6 (46.15%)	7 (53.85%)	0 (0%)
Postnatal	96	24 (25%)	72 (75%)	0 (0%)

B

dpc	No. of embryos	+/+	+/CKO	CKO
9.5	33	9 (27.27%)	17 (51.52%)	7 (21.21%)
10.5	107	17 (15.8%)	68 (63.55%)	22 (20.56%)
11.5	49	12 (24.48%)	28 (57.14%)	9 (18.36%)
12.5	11	3 (27.27%)	6 (54.55%)	2 (18.18%)
13.5	24	9 (37.50%)	14 (58.33%)	1 (4.16%)
Postnatal	17	7 (41.17%)	10 (58.83%)	0 (0%)

Table S1: Analysis of embryo genotypes and lethality mapping.

Table: List of genotyping primers

No	Primer name	Primer sequence (5'- 3')
1	RudGNWTF	TCATGGTGTAGCTAGTGTGG
2	RudGNWTR	CCTGCATTCCGTCACTGTAG
3	RudGNMUTF	AATGGAAGGATTGGAGCTACG
4	RudLoxR2	AATCCCACAACTGCTGCTAC
5	Cre F	ATCCGAAAAGAAAACGTTGA
6	Cre R	ATCCAGGTTACGGATATAGT

Table: List of RT PCR primers

No	Gene	Forward primer (5'- 3')	Reverse primer (5'-3')	Amplicon size (bp)
1	Gapdh	GGTGAGGCCGGTGCTGAG	CCTGCCAAGTATGATGACATC A	514
2	Rudhira	TGCAGCCATGGACGACACAT CACAGAATCTAG	GAAGTGCTCGAGAATGCCATC ACTGTCCGAG	800

Table: List of qPCR primers

No	Gene	Forward primer (5'- 3')	Reverse primer (5'-3')
1	Aebp1	TCCGTATACCTGGGCTGTGA	TGCTCATCTGTCACCACACC
2	Arhgef7	CCTGGACAAGTACCCCACAC	AGCTCCTTCCTCTTGCGAAC
3	Bai1	CCTAATCACTCACTCACCCTC	TGCTTCTCTGGCTTGCTGTC
4	Bai2	CACACTTTCGACCGCTACC	GCCTCCTGTGCTGGGAC
5	Bambi	CTACTGTGATGCTGCCCAC	TGCTTGCAAGAGAGTCCAGG

6	Bmp2	TGGAAAAGGACATCCGCTCC	TCTTGCAGCTGGACTTGAGG
7	Bmp4	GCCATTGTGCAGACCCTAG	ACCCCTCTACCACCATCTC
8	Cldn11	GTGGCTGTTTGCTCTTTCCT	TCAGCAATGTTCCCAAGACA
9	Cntn2	TGGTCTCCTCTCCAGGATGG	GGAATAGCAGGCCAACAGGT
10	Coro1a	AAATTCCGCCACGTGTTTGG	AGTCTTGCCTAGGGGTAGCA
11	Dsg4	GCCACCTTACGGAGTGTTCA	GAGAGCTCGGCAATGGATCA
12	Esm1	CAGTATGCAGCAGCCAAATC	GATGCTGAGTCACGCTCTGT
13	Fgf2	CCAACCGGTACCTTGCTATG	GTCCCGTTTTGGATCCGAG
14	F2r	TGGAGGGTAGGGCAGTCTAC	TACACGGAGGGCATGAAGAG
15	Fos	GGGCTCTCCTGTCAACACAC	CTGGTGGAGATGGCTGTCAC
16	Gapdh	GGTGAGGCCGGTGCTGAG	TGATGTCATCATACTTGGCAGG
17	Hnf4a	GCCTTCTGCGAACTCCTTCT	GCTCATCTCCGCTAGCTCTG
18	Hes5	AACTCCAAGCTGGAGAAGGC	GTCAGGAACTGTACCGCCTC
19	Igfbp7	AAGAGGCGGAAGGGTAAAGC	CTGGGGTAGGTGATGCCG
20	Ins2	GACCCACAAGTGGCACAAC	TCTACAATGCCACGCTTCTG
21	Igf1	CTCTGCTTGCTCACCTTCAC	CACACGAACTGAAGAGCATCC
22	Ins3	CATGCGCGCGCCGCTGCTAC	TCAGTGGGGACACAGACCC
23	Itga4	TATGGCTGCGGAAGCGAG	CACCACCGAGTAGCCAAACA
24	Itgav	GGTGTGGATCGAGCTGTCTT	CAAGGCCAGCATTTACAGTG
25	Itga9	CAGGCCGGAATAGCAGGC	GTGGCCAGCCGTCACTGC
26	Kdr	GCGGAGACGCTCTTCATAAT	CACTTGCTGGCATCATAAGG
27	Kng1	CCTACATTGCCAGGGAGCAA	GCTTTTCATGCAACCAGCCA
28	Mmp-2	CAGGGAATGAGTACTGGGTC	ACTCCAGTTAAAGGCAGCATC
29	Mmp-9	GTCCAGACCAAGGGTACAGC	ATACAGCGGGTACATGAGCG

30	Mmp-10	ATGGACACTTGCACCCTCAG	GTGGAAGTTAGCTGGGCTTG
31	Mmp-21	ACACAGGCATCAGCCTTCTC	AATGACCCCTTGCACGAACC
32	Mmp-25	CTGGCTGTCTGGGCTACTG	GGTAGGCCCGAGCAAAGTG
33	Myh6	TCTGCCTACCTTATGGGGCT	ACTTGCTGTACACTCTGCCC
34	Plat	AGATGAGCCAACGCAGAC	AACTTCGGACAGGCACTG
35	Plg	CGTGGGTTGGATGTTCAGGA	GTCCGGTCAGCAACCATGTA
36	Ppbp	CGTTGTTCCCTCCTGGCTCT	GGACGATGTAGGTCTGAGTC
37	Ptprm	TGTCGGAGCAACCTATCTGTG	CTGGCTGCCGATCATTCCAG
38	rudhira	TCCTACATGGAGAGCGTCG	GGAGGCTCATTTCCAGTGC
39	Serpinb2	TTCCGTGTGAACTCGCATGA	TGCGTCCTCAATCTCATCGG
40	Serpinf2	ACTCTGTACCATCCCTCGCT	ATGTTGCACGCTAGACACCA
41	Smad6	GGGTGAATTCTCAGACGCC	GGTCGTACACCGCATAGAGG
42	Smad7	CCCATCACCTTAGCCGACTC	GCACAGCATCTGGACAGTC
43	Smurf1	AGGAGGGAATTGCAGGTTCG	AGTGAACCTCGCACATCAGG
44	Smurf2	TGGGAAGAAAGGAGAACCGC	CAGCTAAGAGGTCTGCCAGG
45	Sox9	GTGCAAGCTGGCAAAGTTGA	TGCTCAGTTCACCGATGTCC
46	Spi1-3	GAAGCTGCAGCAGTTACAGTC	TGTGGGATCTACCACTTTTCC
47	Tgfb2	TTTAAGAGGGATCTTGGATGG	AGAATGGTCAGTGGTTCCAG
48	Tgfb3	CGCACAGAGCAGAGAATTG	TGACATGGACAGTGGATGC

49	Tgfbr2	CCCAAGTCGGATGTGGAAATG	CGCTGGCCATGACATCACTG
50	Vash1	CCATACCAAGTGTGCCTAC	AGAATTGTGTCCCTGTGTG
51	Vim	GAGAACTTTGCCGTTGAAGC	GCTTCCTGTAGGTGGCAATC
52	Vtn	CCCCTGAGGCCCTTTTTCA	CAAAGCTCGTCACACTGAC
