# Supplementary material for: Rudhira/BCAS3 is essential for mouse development and cardiovascular patterning
Source: Sci Rep. 2018 Apr 4;8:5632. doi: 10.1038/s41598-018-24014-w (PMC5884795; doi:10.1038/s41598-018-24014-w)
Supplement: Supplementary file 4 — S4 [file 41598_2018_24014_MOESM4_ESM.doc]

| **PROCESS** | **DOWN EMB** | **DOWN YS** | **UP EMB** | **UP YS** | **GENES** |
| --- | --- | --- | --- | --- | --- |
| **ANGIOGENESIS / BLOOD VESSEL REMODELLING** | 8 | 8 | 8 | 49 | Ctsh ; Alox12 ; Kdr ; Adora2b ; Sema5a ; Ptgis ; Grem1 ; Foxc2 ; Sema4a ; Plg ; Tgfbr2 ; Lgals3 ; Hoxa5 ; Dcn ; Isl1 ; Nr2e1 ; Thbs2 ; Pik3r6 ; Pgf ; Fgf1 ; Sphk1 ; Cyp1b1 ; Amot ; Crhr2 ; Hipk2 ; Sulf1 ; Hif1a ; C3 ; Sema3e ; Nodal ; Vegfc ; Nf1 ; Map2k5 ; Adamts1 ; Prkca ; Hspb1 ; Notch4 ; Col4a3 ; Srpx2 ; Runx1 ; Prl2c2 ; Ptprm ; Angpt4 ; Ccl5 ; Cd36 ; Ccl24 ; Lep ; Foxc1 ; Hgf ; Lrg1 ; Gdf2 ; Thbs1 ; Ccr5 ; Ngfr ; Lif ; Adm2 ; Ccbe1 ; Angptl3 ; Serpine1 ; Ptn ; Ptgs2 ; Fgf2 ; Uts2 ; Gpr4 ; Il17f ; Ccr3 ; Hdac9 ; Uts2r ; Il1a ; C5ar1 ; Htatip2 ; Cxcl10 ; Apoh ; Prok2 ; Prok1 ; Ccr2 ; Gtf2i ; C3ar1 ; Ntrk1 ; Ccl11 ; Nos3 ; Erap1 ; Vash1 ; Rapgef3 ; Jak1 ; Mmp9 ; Cma1 ; Itgb3 ; Acvrl1 ; Camp ; Enpp2 ; Pde3b ; Ngp ; Flt1 ; Hc ; Chrna7 ; Cx3cr1 ; Sfrp2 ; Dll1 ; Ets1 ; Zc3h12a ; Wnt5a ; Optc |
| **CELL ADHESION** | 50 | 67 | 46 | 331 | Prph2 ; Lcp1 ; Robo2 ; Emb ; Dsp ; Hoxa7 ; Cebpb ; Podxl2 ; Ajap1 ; Cntn6 ; Vwa2 ; Bcl11b ; Fgg ; Abat ; Ret ; Celsr2 ; Hapln1 ; Cntn2 ; Tdgf1 ; Apoa1 ; Ass1 ; Egr3 ; Ttyh1 ; Fga ; Ccdc80 ; Celsr3 ; Vtn ; Dact2 ; Wnt7b ; Col8a2 ; P2ry12 ; Jam2 ; Alox12 ; Cldn2 ; Lyve1 ; Coro1a ; Bcl11a ; Ctnnd2 ; Kdr ; Cd276 ; Hes5 ; Cntn1 ; Ephb6 ; Sema5a ; Grem1 ; Kirrel3 ; Foxc2 ; Postn ; Aplp1 ; Onecut2 ; Il6st ; Sema4a ; Epha3 ; Cxadr ; Plcb1 ; Itch ; Has2 ; Sfrp1 ; Peli1 ; Adamts12 ; Plg ; Wisp1 ; Epdr1 ; Epha7 ; Fstl3 ; Gm1123 ; Efr3a ; Apoa4 ; Tgfbr2 ; Fcer1g ; Lgals3 ; Megf10 ; Pde5a ; Cd24a ; Bcas3 ; Pcdhgc3 ; Tcf7 ; Myh9 ; Col6a2 ; F11r ; Hapln3 ; Ephb1 ; Cbln1 ; Vwf ; Lamb3 ; Wisp2 ; Igsf11 ; Cdh5 ; Fkbp1a ; Satb1 ; Igf1 ; Gpm6b ; Azgp1 ; Pcdh10 ; Celsr1 ; Astn1 ; Nrarp ; Gp1bb ; Lamc3 ; Selplg ; Itga6 ; Mmp24 ; Cdh6 ; Cd4 ; Pgm5 ; Stx3 ; B4galnt2 ; Thbs2 ; Sned1 ; 9430020K01Rik ; Plxnb1 ; Cxcl13 ; Igfals ; Bves ; Ctnna2 ; Pik3r6 ; Itpkb ; Grhl2 ; Tacstd2 ; Il18 ; Tln1 ; Parvb ; Prkg1 ; Wnt4 ; Angpt1 ; Adam12 ; Cyp1b1 ; Malt1 ; Trpv4 ; Serpini1 ; Stab2 ; Cdh3 ; Braf ; Traf6 ; Cdh15 ; Igfbp2 ; Wnt3a ; Hoxd3 ; Cd83 ; Plek ; Cav1 ; Adam23 ; Cxcr4 ; Cd80 ; Pcdh20 ; Chrd ; Ifna1 ; Itga9 ; Kit ; Zc3h12d ; Ikzf1 ; Rsad2 ; Cd200 ; Cdkn2a ; Amica1 ; Cldn11 ; Col7a1 ; Dst ; Cntnap4 ; Dlc1 ; Vav1 ; Lmo1 ; Cass4 ; Adam15 ; Spock1 ; Smoc2 ; Bcl2l11 ; Actb ; Egfl7 ; Clec4g ; Sema3e ; Cdh20 ; Podxl ; Egfr ; Ifna6 ; Bmp2 ; Was ; Ptpru ; Fas ; Itk ; Nodal ; Vegfc ; Cd3e ; Nf1 ; Cldn1 ; Map2k5 ; Tgfb2 ; Ceacam1 ; Sox13 ; Cdh23 ; Relb ; Slit2 ; Lgals7 ; Wnt1 ; Ptprs ; Ncan ; Dscam ; Axl ; Mfi2 ; Sla2 ; Olfm4 ; Slc7a11 ; Nr4a3 ; Prlr ; Nt5e ; Ifnar1 ; Prkca ; Hspb1 ; Dsc2 ; Tsc1 ; Batf ; Cdk6 ; Pcdh8 ; Gsn ; Pnn ; Izumo1 ; Bmp4 ; Fzd7 ; Bmx ; Vit ; Nrxn1 ; H2-DMa ; Pik3cg ; Tcam1 ; Itga2 ; Cdh2 ; Col4a3 ; Myoc ; Bmp7 ; Kitl ; Tinag ; Pip5k1c ; Skap1 ; Spon2 ; Fermt1 ; Nfasc ; Unc13d ; Srpx2 ; Itga11 ; Edil3 ; Rag1 ; Stx11 ; Scarb2 ; Fat3 ; Lgals1 ; Ubash3b ; Ccr7 ; App ; Ebi3 ; Myf5 ; Col14a1 ; Fat4 ; Ache ; Lgals3bp ; Il7 ; Cd44 ; Ceacam2 ; Txk ; Zap70 ; Neo1 ; Vtcn1 ; Zbtb1 ; Il18r1 ; Sdk1 ; Rgmb ; Rpsa ; Ptprm ; Cd28 ; Btnl2 ; Gp5 ; Ccl5 ; Myo1g ; Cdon ; Calca ; Amigo1 ; Ctla4 ; Cd36 ; Il2ra ; Il21 ; Selp ; Ccl19 ; Fgb ; Cdh7 ; F2rl1 ; Il1rl2 ; Wwp1 ; Lat ; S100a8 ; H2-Oa ; Fbln2 ; Il27 ; Scgb1a1 ; Col6a1 ; Lama3 ; Il7r ; Lep ; Kif26b ; Pax1 ; Cntnap5a ; Rapgef1 ; Ephb4 ; Dusp26 ; Bcl10 ; Tnfaip3 ; Col1a1 ; Atp7a ; Mip ; Rasgrp1 ; Pvrl4 ; Mad1l1 ; Cd46 ; Disc1 ; Scarb1 ; Slfn1 ; Il4 ; Lama4 ; Psen1 ; Cd2 ; Col8a1 ; Ptger3 ; Anxa9 ; Emilin1 ; Rs1 ; Trp53 ; Stxbp3 ; Ifna11 ; Lef1 ; Csf3r ; Chl1 ; Strc ; Col12a1 ; Siglec1 ; Epha5 ; Cd96 ; Thbs1 ; Tesk2 ; Adam8 ; Mpzl3 ; Cntn4 ; Bmp10 ; Dscaml1 ; Ifna2 ; Ifna4 ; Ifna5 ; Ifna7 ; Ifna9 ; Ifnb1 ; Ifng ; Ccr5 ; Arhgef7 ; Pcdh12 ; Adora2a ; Nf2 ; Myb ; Myh10 ; Ptprt ; Mill2 ; Tnfsf4 ; Lyn ; Ly9 ; Anxa1 ; Aif1 ; Rltpr ; Cd6 ; Cd84 ; Tnfsf13b ; H2-Aa ; Pcdhb18 ; Pcdhb14 ; Grid2 ; Clec2i ; Angptl3 ; Siglece ; Il23a ; Nlrp3 ; Serpine1 ; Megf11 ; Prkce ; S100a9 ; Npy2r ; Ptn ; Tnn ; Ptprc ; Cdh26 ; Rnase10 ; Clca2 ; Clec4e ; Clec4f ; Ptprk ; Dock2 ; Tnr ; Hepacam ; Ecm2 ; 5830411N06Rik ; Egfl6 ; Btn1a1 ; Slamf1 ; Stat5b ; Reln ; Il15 ; Pdcd1lg2 ; Fzd5 ; Lax1 ; Cd86 ; Tnfsf18 ; Mia3 ; Ctnna3 ; P2rx7 ; Has1 ; Omg ; Gpa33 ; Amtn ; Tbx18 ; Crisp2 ; Ambn ; Pla2g2a ; Unc5d ; Cadm3 ; Spn ; Sit1 ; Dpt ; Fcgr4 ; Foxn1 ; Il4ra ; Klra2 ; Klra5 ; Klra7 ; Agr2 ; Thy1 ; Ptpn2 ; Ptprd ; Rag2 ; Rnd1 ; Vsig4 ; Cbll1 ; Apbb1ip ; Ptpn22 ; Vcam1 ; Cdh8 ; Cdh9 ; Clstn2 ; Tnfrsf18 ; Spam1 ; Hapln4 ; Dsg1a ; Dmp1 ; Dmd ; Cd244 ; Dsc3 ; Nov ; Dsc1 ; Pard3 ; Cadm2 ; Itgad ; Chst4 ; Col13a1 ; Rhoh ; Cd8a ; Cd53 ; Cd22 ; Ptger4 ; Jup ; Itgb7 ; Itgax ; Itgav ; Itgam ; Itga4 ; Crip3 ; Cd226 ; Cd209a ; Ins2 ; Adam2 ; Cd74 ; Hsh2d ; Msln ; Runx2 ; Calr ; Lepr ; Arvcf ; Hapln2 ; Cdh12 ; Fndc3a ; Serpini2 ; Xcl1 ; Trpm7 ; Tnf ; Apc ; Amelx ; Ppara ; Sele ; Eomes ; Alox15 ; Pcdha9 ; Clec4d ; Il1rapl1 ; Lamc2 ; Lama2 ; Spink5 ; Col19a1 ; Cdh17 ; Ccr2 ; Cdh10 ; Ncam2 ; Utrn ; Pag1 ; Bysl ; Bcl3 ; Cntnap5c ; Fat2 ; Lypd3 ; Xrcc4 ; Mkln1 ; Ifna14 ; Gldn ; Csf1 ; Mafb ; Tecta ; Gpnmb ; Ank3 ; Il6 ; Itgb5 ; Acan ; Pdgfra ; Spp1 ; Thbs4 ; Igsf9b ; Foxp1 ; Cytip ; Ifnab ; Eif2ak4 ; Pkp1 ; Negr1 ; Shb ; Lag3 ; Nrxn3 ; Cntn3 ; Rc3h1 ; Cd27 ; H2-Ab1 ; Swap70 ; Muc4 ; Slamf6 ; Vwc2 ; Robo1 ; Il2rg ; Abl2 ; Parvg ; Psmb11 ; Spon1 ; Macf1 ; Sspo ; Flt3 ; Pstpip1 ; Mmp14 ; Adipoq ; Golph3 ; Cdh13 ; Ibsp ; Abi3bp ; Bcan ; Mslnl ; Scarf1 ; Il12rb1 ; Ninj2 ; Pla2g2f ; Cblb ; Hpse ; Klra4 ; Crtam ; Aire ; Itgb3 ; Actn2 ; Casp3 ; Tesc ; Bcl6 ; Src ; Cntn5 ; Myo1f ; Rap2b ; Bcl2a1d ; Ctnnd1 ; Cd1d2 ; Acvrl1 ; H2-M3 ; Ptafr ; Dtx1 ; Sox2 ; Jam3 ; Sp3 ; Npnt ; Fut7 ; Igfbp7 ; Icam5 ; Enpp2 ; Ctla2a ; Rorc ; Glmn ; Pde3b ; Tnfsf9 ; Ptprz1 ; Dsg4 ; Cd2ap ; Dbn1 ; Rell2 ; Chrna7 ; Icam2 ; Pcdhga4 ; Cd3d ; Egflam ; Fblim1 ; Zfp36l2 ; Spock2 ; Mpdz ; Prkcq ; Ephb3 ; Pcdh18 ; Sos2 ; Cx3cr1 ; Klra1 ; Boc ; Plau ; Btla ; Ifna12 ; Dll1 ; Ada ; Pycard ; Cdh16 ; Cdh24 ; Vav3 ; Treml2 ; Gli3 ; Flot2 ; Ptprj ; Sox9 ; Smad7 ; Wnt5a ; Col16a1 ; Cacnb4 ; Btn2a2 ; Psen2 ; Col5a3 ; Itgb6 ; Spaca4 ; Sorbs2 ; Cdh1 ; Pcdh19 ; Tbx21 ; C1qtnf1 ; Comp ; Pcdh15 ; Gm609 ; Flrt1 ; Kirrel2 ; Azi2 |
| **CELLULAR RESPONSE TO ESTROGEN STIMULUS** | 3 | 4 | 1 | 7 | Sfrp1 ; Sstr2 ; Bcas3 ; Crhbp ; Naip2 ; Egfr ; Hsf1 ; Sstr1 ; Myod1 ; Esr1 ; Ncoa3 ; Msx2 ; Nrip1 ; Myog ; Trim24 ; Ramp3 |
| **CYTOSKELETON** | 60 | 65 | 46 | 295 | Nefm ; Kbtbd8 ; Lcp1 ; 2900011O08Rik ; Abra ; Dsp ; Pmp22 ; Krt19 ; Kif1b ; Gemin5 ; Timeless ; Plk3 ; Ccdc116 ; Cntn2 ; Cotl1 ; Cyp27a1 ; Mid1 ; Klc4 ; Fos ; Lmx1b ; Apob ; Coro1a ; Plek2 ; Wdr66 ; Kdr ; Mlph ; Hap1 ; Stmn4 ; Dhcr24 ; Fzd10 ; Tubb6 ; Ina ; Nwd1 ; Synpo ; Kif5a ; Keg1 ; Rif1 ; Sncg ; Tcf15 ; Pcm1 ; Mtrr ; Stmn2 ; Onecut2 ; Epha3 ; Cxadr ; Wdfy1 ; Sfi1 ; Zfp365 ; Ppl ; Sfrp1 ; Prc1 ; Vil1 ; Baiap2l1 ; Mark1 ; Kcnab2 ; Bin3 ; Nup37 ; Cep72 ; Stk38l ; Ahnak ; Tppp3 ; Plekhg6 ; Kif18a ; Itpka ; Crmp1 ; Tagln3 ; Bsn ; Ptpn14 ; Bcas3 ; Mapt ; Nostrin ; Llgl2 ; Arhgap24 ; Bub3 ; Enah ; Stmn3 ; Cobl ; Myh9 ; Tubal3 ; Kif2b ; Smpx ; F11r ; Myoz2 ; Hydin ; Mvp ; Cdc40 ; Nr3c1 ; Eps8 ; Slc16a3 ; Kif5c ; Cep97 ; Edn1 ; Zfp185 ; Afap1 ; Prkci ; Cdh5 ; Acta2 ; Iqgap1 ; Vim ; Nek6 ; Gpm6b ; Capn2 ; Herc1 ; Nsmaf ; Celsr1 ; Rbbp6 ; Sh3kbp1 ; Dnaic1 ; Rttn ; Fam110c ; Ttn ; Rapgef5 ; Scap ; Fez1 ; Pwp1 ; Shroom4 ; Basp1 ; Pgm5 ; Smek2 ; Fam101a ; Cntln ; Strbp ; Aldob ; Plxnb1 ; Rhof ; Invs ; Ky ; Cxcl13 ; AI314180 ; Ctnna2 ; Odf2 ; Arc ; Larp4 ; Nr1i3 ; Rasgrp3 ; Rasgef1c ; Ikbkb ; Rph3a ; Dclk2 ; Add2 ; Ppp2r2b ; Tln1 ; Parvb ; Elavl4 ; Dpysl2 ; Wdr17 ; 4931431F19Rik ; Tacc2 ; Amot ; Amph ; Trpv4 ; Ntf3 ; Ldb3 ; Dmxl2 ; Spag4 ; Braf ; Wnt3a ; Plod3 ; Plek ; Pou6f1 ; Cav1 ; Ttll4 ; Dpysl3 ; Fbxl7 ; Pdgfrb ; Grhl3 ; Cabyr ; Akap12 ; Tradd ; Acta1 ; Evc ; Kit ; Ssh3 ; Ptgfrn ; Nanog ; Ccdc155 ; Ldb2 ; Hspb7 ; Dst ; Dlc1 ; Fscn3 ; Tnnc1 ; Cass4 ; Pdxp ; Fhl3 ; Fgf10 ; Rbbp7 ; Cep70 ; Msn ; Tmod2 ; Frmd8 ; Rpgr ; Myom2 ; Actb ; Sema3e ; 1110017D15Rik ; Egfr ; Was ; Capn6 ; Tns4 ; Ftcd ; Gzmb ; Myo3b ; Itk ; 2700060E02Rik ; Frmd4a ; 1700029J07Rik ; Krt13 ; Taf1a ; Rad18 ; Fyb ; Nf1 ; Prepl ; Tgfb2 ; Wdr5b ; Tubb3 ; Avil ; Relb ; Sprr1b ; Wdr90 ; Nin ; Plxna3 ; Ptprs ; Slc7a11 ; Slc30a9 ; Spef1 ; Mastl ; Prkca ; Hspb1 ; Sh2d2a ; Cntrob ; Rapsn ; Tsc1 ; Cdk6 ; Spry2 ; Gsn ; Lats1 ; Kif9 ; Polr2c ; Fgd2 ; Cep250 ; Fhod3 ; Itga2 ; Nav1 ; Birc6 ; Cdh2 ; Fgd4 ; Kitl ; Pip5k1c ; Chd3 ; Svil ; Fermt1 ; Spag16 ; Myo5a ; Slc2a4 ; Ern1 ; Nfasc ; Itga11 ; Rap2a ; Tnik ; Nek7 ; Mapk9 ; Tppp ; Trip4 ; Spag6 ; Fscn2 ; Ptpn21 ; Ccr7 ; Son ; Kif20a ; Fgd6 ; Fmn2 ; Pfdn2 ; Pfn3 ; Zfyve19 ; Synpo2 ; Sgca ; Dnm1 ; Gys2 ; Cd44 ; Krt84 ; Kars ; Sntb1 ; Pacsin2 ; Pak7 ; Ophn1 ; Katnal2 ; Lats2 ; Ptpn20 ; Coro2a ; Ivns1abp ; Kif13b ; Sdk1 ; D7Ertd443e ; Atp8a2 ; Gtf2f2 ; Nod2 ; Katnb1 ; Lmod1 ; Lor ; Myo1g ; Krt27 ; Scyl1 ; Ulk4 ; Cd36 ; Becn1 ; Plk4 ; Selp ; Arhgef19 ; Ttl ; Wdr48 ; Rhbg ; Lat ; Ccl24 ; S100a8 ; Csrp3 ; Ankra2 ; Camk2b ; Arhgef5 ; Dmpk ; Ubxn11 ; Lrrk2 ; Arl2bp ; Kif26b ; Pacsin1 ; Btk ; Anxa11 ; Ctsl ; Racgap1 ; Rapgef1 ; Syne2 ; Sipa1l3 ; Usp44 ; Sac3d1 ; Btrc ; Cacna1c ; Rasgrp1 ; Bop1 ; Armc4 ; Mad1l1 ; Wasf1 ; Htr2a ; Disc1 ; Sgcd ; Vprbp ; Ccnf ; Sntg1 ; Psen1 ; Neil2 ; Nbeal1 ; Tmem67 ; Wdr82 ; Dock4 ; Trpc6 ; Dgkq ; Zmynd10 ; Strn ; Trp53 ; Dmd ; Tnf ; Apc ; Ifng ; Gfap ; Nefl ; Nf2 ; Fcgr2b ; Itgam ; Reln ; Prkce ; Calb1 ; Tbce ; Nlrp3 ; Fgr ; S100a9 ; Il1a ; Myh10 ; Dock2 ; Thy1 ; Jup ; Tlr3 ; Sele ; Tagln ; Ncf1 ; Calr ; Pard3 ; Anxa1 ; Snap25 ; Kcnma1 ; Itgav ; Grm5 ; Ccna1 ; Rasgrf1 ; Mtss1 ; Itgax ; Krt16 ; Krt71 ; Tmsb4x ; Ccr1 ; Aif1 ; Tnfrsf11a ; Myo15 ; Adcy10 ; Kif17 ; Trpc4 ; Cckar ; Pon1 ; Hira ; Pstpip2 ; Alox8 ; Ptpn13 ; Syne1 ; Epha5 ; Gnai1 ; Cenpe ; Nbea ; Nek1 ; Nek2 ; Sgce ; Nphs2 ; Rangap1 ; Myh4 ; Krt4 ; Trpm4 ; Slpi ; Myo1e ; Cdk7 ; Gas2 ; Myo1a ; Arhgef7 ; Rgs14 ; Efhc1 ; Nusap1 ; Wee1 ; Ehd2 ; Coro6 ; Wdr19 ; Padi6 ; Ctnna3 ; Klk6 ; Rasgrf2 ; Dnm3 ; Ms4a2 ; Fmnl1 ; Dsg1a ; Tep1 ; Ptpn4 ; Ptpn3 ; Synj2 ; Zfp106 ; Fbxw2 ; Nrap ; Apbb1ip ; Klhl1 ; Myo3a ; Ablim2 ; Ttll10 ; Bcar3 ; Eml5 ; Ttll8 ; Akap4 ; Inpp5e ; Wdr36 ; Trim9 ; Capza3 ; Rp1l1 ; Ptpn7 ; Mefv ; Stxbp4 ; Ttll6 ; Usp33 ; Dsc3 ; Odam ; Insrr ; Itgad ; Map6d1 ; Stox1 ; Actl7b ; Orc4 ; Rnd1 ; Wdr27 ; Ablim3 ; Mdn1 ; Lrrcc1 ; Wdr44 ; Frmpd1 ; Sgcz ; Kif24 ; Grid2ip ; Wdr41 ; Spag17 ; Wdr12 ; Ccdc39 ; Iqsec3 ; Actrt1 ; Rltpr ; Cep76 ; Wdr18 ; Ermn ; Actbl2 ; S100a14 ; Ofcc1 ; Ubqln3 ; Lrrc26 ; Ppwd1 ; Fbxw10 ; Pcdhb5 ; Cdc20b ; Ddx60 ; Tesk2 ; Sec31b ; Zfp174 ; Wdr88 ; Ccdc87 ; Ubqlnl ; Kncn ; Slc12a5 ; Utrn ; Sod1 ; Pag1 ; Ccdc14 ; Frmd7 ; Ccdc38 ; Iqsec2 ; Snca ; Gpc3 ; Actl7a ; Psmb3 ; Phf1 ; Kif12 ; Mkln1 ; Tuba8 ; Wdr72 ; Ccl11 ; Ank3 ; Gnb3 ; Plekhh2 ; Sntg2 ; Nos3 ; Pclo ; Nos1 ; Ugt8a ; Nfatc2 ; Rapgef3 ; Jak1 ; Ins1 ; Sgcg ; Ssh1 ; Rgl3 ; Swap70 ; Zfp207 ; Lsp1 ; Mmp9 ; Abl2 ; Parvg ; Neil1 ; Herc2 ; Agbl2 ; Clic5 ; Kifc2 ; Macf1 ; Phc3 ; Arhgef17 ; Ppp4r2 ; Akt1 ; Sox17 ; Mpp1 ; Pstpip1 ; Stim1 ; Ofd1 ; Rasgrp4 ; Csf1r ; Spatc1 ; Adipoq ; Trim29 ; Scin ; Fry ; Baiap2l2 ; Daam1 ; Ift57 ; Ksr2 ; Gas2l2 ; Actrt2 ; Ints6 ; Ss18 ; Kif26a ; Igf2bp2 ; Nbeal2 ; Cap2 ; Zmym4 ; Kif3b ; Sp4 ; Tekt3 ; Itgb3 ; Spata7 ; Actn2 ; Casp3 ; Bcl6 ; Synpo2l ; Traf3ip1 ; Ccdc113 ; Src ; Wdr35 ; Tmod4 ; Myo1f ; Ntrk3 ; Nphp1 ; Vangl2 ; Kif5b ; Sdccag8 ; Npm1 ; Ccdc92 ; Myo7b ; Brsk1 ; Jam3 ; Zranb1 ; 4921507P07Rik ; Dixdc1 ; Tyrobp ; Pfn4 ; Dlg4 ; Arhgef10l ; Apc2 ; Dynlrb2 ; Bbs1 ; Lrp1 ; Wdr13 ; Fgf7 ; Dsg4 ; Kif1a ; Snta1 ; Cd2ap ; Dbn1 ; Flt1 ; Phip ; Pik3r4 ; Fgd3 ; Ccdc96 ; Ckap2 ; Fblim1 ; Lpin1 ; Frmpd4 ; Rapgef4 ; Irs1 ; Prmt1 ; Myoz1 ; Ush1g ; Mapk8 ; Camk2a ; Kif21b ; Sos2 ; Prkaa2 ; Trim67 ; Lrrc48 ; Pak6 ; Pdlim3 ; Eml1 ; Gpr65 ; Mak ; Sh2b2 ; Klhl2 ; Tle2 ; Dctn4 ; Kif1c ; Trib2 ; Sh2d3c ; Ttll11 ; Snph ; Map2k7 ; Flot2 ; Hook3 ; Nos2 ; Sox9 ; Ccdc85b ; Zc3h12a ; Dctn3 ; Wipf1 ; Ttc21b ; Hepacam2 ; Mapk6 ; Mapkbp1 ; Krt24 ; Sorbs2 ; Cdh1 ; Cttnbp2nl ; Uxt ; Tle1 ; Ctsd ; Gulp1 ; Nfkb1 ; Hoxc8 ; Dcdc2a ; Apbb3 ; Fam110b |
| **ECM DEGRADATION** | 8 | 8 | 2 | 33 | Fos ; Tnfrsf11b ; Tac1 ; Kdr ; Fmod ; Plg ; Blnk ; Adamts7 ; Apoe ; Adcyap1 ; Met ; Fgf1 ; Tln1 ; Pik3cd ; Wnt3 ; Cav1 ; Mme ; Bcl2l11 ; Hif1a ; Egfr ; Fas ; Gzmb ; Lgmn ; Tlr4 ; Wnt1 ; Itga2 ; Wnt11 ; Epas1 ; Lrp5 ; Angptl4 ; App ; Casp14 ; Mepe ; Fbn1 ; Mmp10 ; Mep1b ; Ctsl ; Col1a1 ; Atp7a ; Cnr2 ; Mmp12 ; Reln ; Tnf ; Runx2 ; Ptgs2 ; Serpine1 ; Mmp3 ; Itgam ; Fgf2 ; Tnfrsf1b ; Mmp8 ; Dmp1 ; Tlr3 ; Calr ; Lpl ; Mcpt4 ; Cd209a ; Bambi ; Angptl2 ; Sod1 ; Gpnmb ; Il6 ; Pgr ; Pdgfra ; Spp1 ; Mmp9 ; Hoxa11 ; Abl2 ; Eln ; Akt1 ; Cma1 ; Mmp14 ; Adipoq ; Hpse ; Src ; Lrp1 ; Ctsk ; Mapk8 ; Trpv1 ; Cst3 ; Col17a1 ; Smad7 ; Wnt5a ; Ctsd |
| **ESTABLISHMENT OR MAINTENANCE OF EPITHELIAL CELL APICAL/BASAL POLARITY** | 1 | 0 | 4 | 8 | Lin7a ; Tcf15 ; Lin7b ; Cdx2 ; Crb2 ; Prickle2 ; Msn ; Wnt11 ; Vangl2 ; Dlg4 ; Arf4 ; Wnt5a |
| **MORPHOGENESIS** | 63 | 61 | 76 | 369 | Tbx20 ; Hes3 ; Heyl ; Dcx ; Hoxc9 ; Dsp ; Hoxa7 ; Ski ; Ctsh ; Bcl11b ; Timeless ; Hoxa1 ; Folr1 ; Stk3 ; Ret ; Celsr2 ; Sall1 ; Cntn2 ; Tdgf1 ; Cdx4 ; Phlda2 ; Hoxb1 ; Hoxd10 ; Alx3 ; Fst ; Ptf1a ; Tasp1 ; Lmx1b ; Dact2 ; Plxna4 ; Wnt7b ; Col8a2 ; Apob ; Tmie ; Tbx2 ; Wnt2 ; Gcg ; Sst ; Ctnnd2 ; Kdr ; Serpinf2 ; Hmga2 ; Chrna3 ; Hap1 ; Hes5 ; Rab25 ; Myh7 ; Cntn1 ; Aldh1a2 ; Wnt8b ; Lhx4 ; Sema5a ; Grem1 ; Kirrel3 ; Osr1 ; Foxc2 ; Postn ; Nkx3-1 ; Tcf15 ; Pcm1 ; Foxh1 ; Onecut2 ; Il6st ; Epha3 ; Wnt6 ; Cxadr ; Fus ; Hand1 ; Col5a2 ; Zfp365 ; Lhx1 ; Slc6a4 ; Bmpr1b ; Pax4 ; Hoxb6 ; Has2 ; Sfrp1 ; Cdx1 ; Adamts12 ; Hoxd4 ; Vil1 ; Hoxb7 ; Chrnb2 ; Rbp4 ; T ; Pou4f1 ; Foxa3 ; Pou3f4 ; Epha7 ; Sfn ; Dcc ; Shank3 ; Cdx2 ; Ahnak ; Rab23 ; Rspo3 ; Sema3a ; Hoxa9 ; Tgfbr2 ; Ephb2 ; Itpka ; Prrx1 ; Lgals3 ; Hoxa5 ; Cd24a ; Llgl2 ; Tcf7 ; Dcn ; Myh9 ; Apoe ; Esx1 ; Fgfr3 ; Ephb1 ; Actn3 ; Six4 ; Fgf18 ; Nr3c1 ; Eps8 ; Edn1 ; Isl1 ; Lamb3 ; Sox3 ; Prkci ; Cryaa ; Nr2e1 ; Fkbp1a ; Ripply1 ; Satb1 ; Vim ; Sox11 ; Foxq1 ; Osr2 ; Neurog1 ; Tead2 ; Igf1 ; Neurog3 ; Tsku ; Dpysl4 ; Celsr1 ; Pik3ca ; Cldn4 ; Prrx2 ; Sox8 ; Ttn ; Lamc3 ; Gja5 ; Itga6 ; Gata5 ; Mmp24 ; Fgf9 ; Adra2a ; Ghrl ; Jarid2 ; Hoxd8 ; Htr2b ; Dvl3 ; Tbx4 ; Col2a1 ; Shroom4 ; Fam101a ; Wnt9a ; Dclk1 ; Lhx9 ; Rspo2 ; Cited1 ; Plxnb1 ; Ripply2 ; Id4 ; Esrrb ; Sema3c ; Myf6 ; Met ; Vdr ; Ctnna2 ; Rfx4 ; Maf ; Arc ; Larp4 ; Zfp157 ; Myh6 ; Foxp2 ; Pgf ; Gal ; Grhl2 ; Fgf1 ; Gfra1 ; Gpm6a ; Tacstd2 ; Nbl1 ; Hipk1 ; Gbx2 ; Sall3 ; Tln1 ; Stra6 ; Glce ; Dkk1 ; Wnt4 ; Elavl4 ; Neurod1 ; Pdgfc ; Klk14 ; Angpt1 ; Shox2 ; Cyp1b1 ; Amot ; Drd2 ; Sp5 ; Treh ; Cdkl5 ; Slc8a1 ; Foxd2 ; Wwox ; Snx2 ; 2410089E03Rik ; Stab2 ; Cdh3 ; Braf ; Cyp7b1 ; Traf6 ; Wnt3a ; Wnt3 ; Hoxd3 ; Plod3 ; Plekho1 ; Sfrp5 ; Mgp ; Nkx2-1 ; Mmp16 ; Slc9a6 ; Sftpb ; Cacna1f ; Fli1 ; Col1a2 ; Cxcr4 ; Hey2 ; Pdgfrb ; Grhl3 ; Ncoa2 ; Tshz3 ; Cul1 ; Itga9 ; Ovol2 ; Six2 ; Man2a1 ; Kit ; Nfib ; Pla2g3 ; Chsy1 ; Dlc1 ; Tnnc1 ; Hipk2 ; Sulf1 ; Pax6 ; Nog ; Fgf10 ; Bcl2l11 ; Sgk3 ; Trps1 ; Msn ; Caprin2 ; Flt4 ; Ryr2 ; Rpgr ; Hif1a ; Slc26a4 ; Sh3gl2 ; Dnmt3a ; Slitrk6 ; Sp8 ; Egfr ; Bmp2 ; Slit3 ; Mylk2 ; Tlx2 ; Wnt9b ; Sox10 ; Nodal ; Mapk8ip2 ; Rpe65 ; Vegfc ; Lgr5 ; Nphp3 ; Nf1 ; Tgfb2 ; Thra ; Ptch1 ; Zic3 ; Etv4 ; Cdh23 ; Avil ; Wnt8a ; Nin ; Slit2 ; Serpinh1 ; Tlr4 ; Wnt1 ; Dscam ; Wnt2b ; Lefty1 ; Adamts1 ; Hoxc10 ; Tmed2 ; Gak ; Chrna9 ; Nr4a3 ; Gsx2 ; Prkca ; Rln1 ; Bsg ; Pcdh8 ; Tgfbr3 ; Spry2 ; Gsn ; Pnn ; Lats1 ; Vax2 ; Bmp4 ; Notch4 ; Itga2 ; Cdh2 ; Lhx6 ; Klf5 ; Bmp7 ; Wnt11 ; Tbx5 ; Epas1 ; Lrp5 ; Unc93b1 ; Foxl1 ; Rpgrip1 ; Rap2a ; Runx1 ; Tnik ; Foxd1 ; Foxb2 ; Mapk9 ; Fat3 ; Slit1 ; Slitrk5 ; Plag1 ; Rax ; Myf5 ; Pitx2 ; Hand2 ; Fat4 ; Gas7 ; Ache ; Sp1 ; Sox5 ; Satb2 ; Jag1 ; Il7 ; Lrp8 ; Cd44 ; Tbx15 ; Zap70 ; Chst11 ; Pacsin2 ; Lmbr1 ; Hoxd9 ; Foxo1 ; Trpc5 ; Lats2 ; Cd247 ; Sdk1 ; Cacna1a ; Atp8a2 ; Tiparp ; Ereg ; Fbn1 ; Mapk8ip3 ; Ifitm5 ; Arid5b ; Krt27 ; Cdon ; Stc1 ; Ulk4 ; Fgf4 ; Noto ; Gjb6 ; Gjb5 ; Mmp10 ; Wdr48 ; Npy5r ; Camk2b ; Prdm1 ; Naglu ; Btbd7 ; Il7r ; Lrrk2 ; Lep ; Pax1 ; Gprc6a ; Nox4 ; Pacsin1 ; Krt25 ; Ctsl ; Foxo6 ; Foxc1 ; Fgf3 ; Ephb4 ; Sipa1l3 ; Btrc ; Mtnr1b ; Col1a1 ; Atp7a ; Hgf ; Mmp28 ; Foxd4 ; Wasf1 ; Tmem100 ; Pax2 ; Mesp1 ; Disc1 ; Crygs ; Mtnr1a ; Hoxd12 ; Mdfi ; Col9a1 ; Smtnl1 ; Mmp25 ; Hoxd11 ; Mycn ; Bcl2l2 ; Psen1 ; Btc ; Tmem67 ; Cspg5 ; Mmp12 ; Ep300 ; Col8a1 ; Slain1 ; Dlgap1 ; Dlx1as ; Trpc6 ; Il10 ; Pax8 ; Trp53 ; Trp63 ; Tnf ; Mef2c ; Crb1 ; Hoxd13 ; Esr1 ; Runx2 ; Reln ; Foxn1 ; Fgf2 ; Lif ; Lef1 ; P2rx7 ; Ngfr ; Tcf7l2 ; Thbs1 ; Adora2a ; Cybb ; Fmr1 ; Hoxa3 ; Lgr4 ; Nrg1 ; Vcam1 ; Thrb ; Atp2b2 ; Foxo3 ; Prop1 ; Itgav ; Atoh1 ; Erbb4 ; Cxcl10 ; Tbx18 ; Aldh1a1 ; Nefl ; Grid2 ; Ace ; Eomes ; Npy2r ; Runx3 ; Aldh1a3 ; Lcn2 ; Mmp3 ; Nrp2 ; Areg ; Akt2 ; Pitx3 ; Six3 ; Ncoa1 ; Cntf ; Tshr ; Hoxa10 ; Fzd5 ; Emx2 ; Myh10 ; Adam8 ; Aqp2 ; Gaa ; Sox6 ; Nf2 ; Wdr19 ; Mmp8 ; Mtss1 ; Pth ; Gcm1 ; Edar ; Ltf ; Serpinb5 ; Bmp10 ; Epha5 ; Myo15 ; Grk1 ; Gdf2 ; Pcsk5 ; Dmrt1 ; Arhgef7 ; Tlx1 ; Hmx3 ; Prok2 ; Gab2 ; Tead1 ; Chl1 ; Enam ; Angptl3 ; Evx2 ; Mtm1 ; Krt6a ; Mmp20 ; Syne1 ; Matn1 ; Krt71 ; Sycp2 ; Wee1 ; Ptprd ; Krt16 ; Crygb ; Agr2 ; Dnm3 ; Slc4a10 ; Mixl1 ; Gsdma3 ; Nlrp5 ; Dhrs3 ; Slc39a1 ; Kif17 ; Prdm14 ; Foxi1 ; Krt6b ; Cngb1 ; Uty ; Fgf20 ; Grxcr1 ; Dkk4 ; Il1rapl1 ; Klk1b1 ; Npas1 ; Bcar3 ; Mael ; Caps2 ; Gcnt3 ; Nrg3 ; Arhgef6 ; Rnase10 ; Pspn ; Capza3 ; Trip11 ; Rasip1 ; Mmp21 ; Chrna10 ; Dscaml1 ; Rp1l1 ; Stox1 ; Mmp1b ; Mmp27 ; Zpbp ; Ermn ; Actl7b ; Gje1 ; Nubpl ; Gna12 ; Ltbp3 ; Ikbkap ; Plxdc1 ; Kcnq4 ; Gpc3 ; Actl7a ; Sp9 ; Csf1 ; Mafb ; Adamts16 ; Gli1 ; Ccl11 ; Vsx2 ; Esr2 ; Ptpro ; Il6 ; Myh14 ; Pgr ; Nell1 ; Nos3 ; Pdgfra ; Ugt8a ; Rfx3 ; Hoxb8 ; En1 ; Thbs4 ; Nek3 ; Foxg1 ; Casr ; Baz1b ; Gdf5 ; Ar ; Shb ; Cav3 ; Foxe1 ; Ncoa3 ; Frzb ; Ssh1 ; Ssbp1 ; Rxfp1 ; Upk3a ; Pbx1 ; Hmx2 ; Mmp9 ; St14 ; Hoxa11 ; Abl2 ; Robo1 ; Agtr2 ; Ahr ; Alx4 ; Sox17 ; Hoxa6 ; Atp6v1b1 ; Ofd1 ; Mmp14 ; Msx2 ; Ndp ; Vpreb1 ; Fry ; Daam1 ; Ift57 ; Als2 ; Otx2 ; Ss18 ; Jmjd1c ; Hpse ; Tbcc ; Cap2 ; Zmym4 ; Foxl2 ; Tekt3 ; Xlr4b ; Itgb3 ; Insr ; Bcl6 ; Traf3ip1 ; Notch2 ; Ntn4 ; Src ; Cebpa ; Pou4f3 ; Gas1 ; Zfpm2 ; Vangl2 ; Ptprq ; Kiss1r ; Ctnnd1 ; Acvrl1 ; Brsk1 ; Hoxb5 ; Prkar1b ; Rnf165 ; Sox2 ; Grin1 ; Zranb1 ; Vsig1 ; Sp3 ; Twist2 ; Zswim6 ; Hesx1 ; Npnt ; Aqp5 ; Dlg4 ; Tnni3 ; Crispld1 ; Ntf5 ; Enpp2 ; Nfatc1 ; Bbs1 ; Lrp1 ; Ptprz1 ; Fgf7 ; Zic2 ; Dbn1 ; Flt1 ; Phip ; Chrna7 ; Akp3 ; Trp73 ; Mmp23 ; Mst1r ; Vpreb2 ; Frmpd4 ; Mdm4 ; Tcap ; Prom1 ; Foxr1 ; Itgb8 ; Ephb3 ; Ush1g ; Artn ; Mapk8 ; Chrna5 ; Meis1 ; Eya1 ; Hoxc13 ; Gdnf ; Sfrp2 ; Prox1 ; Hoxb13 ; 1110004E09Rik ; Dll1 ; Hey1 ; Casz1 ; Iqub ; Tgfb3 ; Mnx1 ; Egf ; Six1 ; Pax3 ; Igfbp5 ; Map3k14 ; Sp7 ; Dlx5 ; Emx1 ; Gli3 ; Ctnnbip1 ; Col11a1 ; Crispld2 ; Sox9 ; Arhgap5 ; Smad7 ; Alx1 ; Wnt5a ; Th ; Gdf11 ; Psen2 ; Plxna1 ; Ttc21b ; Myl3 ; Hes7 ; Tgfa ; Ndst1 ; Cdh1 ; Krt17 ; Pax9 ; Rtn4 ; Cacna1s ; Pcdh15 ; Ctsd ; Nfkb1 ; Hoxc8 ; Dcdc2a |
| **PEPTIDASE ACTIVITY** | 46 | 46 | 31 | 218 | Itih2 ; Thsd4 ; Serpina1d ; Ctsh ; Ret ; Pappa ; Serpinb13 ; Pcolce2 ; Gzmd ; Serpina1b ; Wfdc1 ; Tasp1 ; Serpinb1a ; Pcsk9 ; Vtn ; Ambp ; Proc ; Alox12 ; Serpinf2 ; Serpina10 ; BC048546 ; Dhcr24 ; Anpep ; Pcsk2 ; Ctsc ; Ggh ; Cfi ; Cpm ; Tmprss7 ; Adam32 ; Cpa2 ; Cpa1 ; Nkx3-1 ; C2 ; Psmb1 ; Plat ; Gzmg ; Sfrp1 ; Prss23 ; Adamts12 ; Cpb1 ; Serpina1a ; Vil1 ; Htra1 ; Prss35 ; Plg ; Kng1 ; Epha7 ; Sfn ; Serpina1c ; Wfdc2 ; F10 ; Serpinb9f ; Scrn1 ; F5 ; Adamts7 ; Otud4 ; Ggt7 ; Cts3 ; Ctsj ; Ctla2b ; Pgc ; Rhbdl3 ; Ren1 ; Adamtsl2 ; Dpep1 ; Cpa5 ; Crb2 ; Serpinb9g ; Cryaa ; Prtn3 ; Tmprss11c ; Cst9 ; Igf1 ; F7 ; Capn2 ; Kng2 ; Serpind1 ; Nradd ; Usp26 ; Mmp24 ; Adamts2 ; Wnt9a ; Cpz ; Agtpbp1 ; Npepps ; Ky ; Bcl2l12 ; Naip2 ; Serping1 ; Adamtsl4 ; Ecel1 ; Usp19 ; Klk14 ; Adam12 ; Pik3ip1 ; Malt1 ; Prss22 ; Serpini1 ; Vcpip1 ; Tpp2 ; F2 ; Wnt3a ; Spink2 ; Hip1 ; Adamtsl1 ; Cav1 ; Mmp16 ; Adam11 ; Adam23 ; C1rl ; Capn8 ; Agbl1 ; Serpinb12 ; Dpp6 ; Ctrc ; Mbtps1 ; Cdkn2a ; Col7a1 ; Dlc1 ; Itih5 ; Asprv1 ; Mme ; Adam15 ; Spock1 ; Bcl2l11 ; Proz ; Itih3 ; Naaladl1 ; Usp29 ; C3 ; Serpinb6b ; Siah2 ; Fas ; Capn6 ; Gzmb ; Timp4 ; Prss3 ; Nodal ; Prepl ; Map2k5 ; Lgmn ; Napsa ; Htra3 ; H13 ; Serpinh1 ; Adamts1 ; Hgfac ; Gsn ; Crim1 ; Nod1 ; Bok ; F2r ; Serpina7 ; Birc6 ; Tfpi ; Col4a3 ; Tmprss13 ; Map3k5 ; Klk1b4 ; Eif2ak3 ; Rag1 ; Mapk9 ; Adamts13 ; App ; BC051665 ; Phex ; Casp14 ; Serpina1e ; Cd44 ; Adamts19 ; Cfd ; Ctsw ; Cst12 ; Hp ; Gzme ; Stfa1 ; Mmp10 ; Cpxm2 ; S100a8 ; Adam18 ; Wfdc6a ; Psmb9 ; Prss16 ; Mep1b ; Capn11 ; Cntnap5a ; Ctsl ; Ctrb1 ; Serpinb6c ; Il24 ; Usp44 ; Birc7 ; Otud1 ; Bcl10 ; Tnfaip3 ; Spock3 ; Hgf ; Mmp28 ; Klk13 ; Tpsab1 ; Cd46 ; Pga5 ; Serpinb9e ; Tmprss3 ; Pax2 ; F8 ; Mmp25 ; 1810009J06Rik ; Itih4 ; Psen1 ; Prss29 ; Mmp12 ; Ep300 ; Dhh ; Serpina3m ; Ctsg ; Acrbp ; Adamts8 ; Hspa1b ; Serpinb9d ; Wfdc3 ; Usp34 ; Hsf1 ; Spink6 ; Lef1 ; Serpinb9 ; Ctse ; Adam7 ; Thbs1 ; Psma1 ; Adam8 ; Ovch2 ; Casp4 ; Naalad2 ; Nr4a1 ; Cst8 ; Adam28 ; Adam25 ; F11 ; Adora2a ; Tll2 ; Serpinb1c ; Klk5 ; Serpina3b ; Serpina3f ; Klk1b11 ; Klk1b21 ; Klk1b5 ; Klk1b1 ; Klk1b8 ; Ctsm ; Mep1a ; Klk12 ; Mcpt4 ; Adamts14 ; Tmprss9 ; Serpina3a ; Serpinb5 ; Ltf ; Mmp21 ; Try10 ; Pcsk5 ; Pcsk1 ; Capn9 ; Klk6 ; Fetub ; 1700074P13Rik ; Mmp3 ; Tmprss11e ; Serpina3g ; Serpinb3d ; Aebp1 ; Aph1c ; Nlrp3 ; Cpa6 ; Serpine1 ; Uchl4 ; 2210010C04Rik ; Tnfsf15 ; S100a9 ; Rbp3 ; Spint4 ; Mmp8 ; Casp12 ; Clca2 ; Nrip2 ; Adam21 ; Nlrp2 ; Usp15 ; Fabp1 ; Reln ; Usp25 ; Tfpi2 ; Usp33 ; Serpinb7 ; Serpinb3b ; Folh1 ; Cradd ; Cflar ; Serpinb2 ; Serpinb3a ; Mug2 ; Pparg ; Taf2 ; Htra4 ; Wap ; Trhde ; Mmp1b ; Nlrp12 ; Usp14 ; Prss28 ; Cyct ; Pmaip1 ; Casp8ap2 ; Capns2 ; Mgmt ; Usp9x ; Klk1b9 ; Cpvl ; 4930444G20Rik ; Tll1 ; Usp53 ; Klk4 ; Usp50 ; Mmel1 ; Serpinb9b ; Gzmc ; Wfdc15b ; Serpinb10 ; Stfa2 ; Cma2 ; Unc5cl ; F9 ; Mmp20 ; Wfdc12 ; Usp27x ; Masp2 ; Trp63 ; Klk9 ; Wfdc6b ; Adam2 ; Adam29 ; Scrn3 ; Mug1 ; Rest ; Akt2 ; Ctsq ; Mefv ; Klkb1 ; Serpina3c ; Prss32 ; Serpini2 ; Serpinb1b ; Tnf ; Slpi ; Adam5 ; Dpep3 ; Cts8 ; Wfdc13 ; Acr ; Ddi1 ; Ace ; Pzp ; Klk11 ; Tmprss6 ; Dpp10 ; Adam22 ; Spink5 ; Tmem27 ; Klk10 ; Psma8 ; Avp ; F12 ; Prss12 ; Serpina6 ; Capn13 ; Snca ; Psmb3 ; Gpc3 ; Egfbp2 ; Spink10 ; Adamts16 ; Igbp1 ; Try4 ; Ctss ; Il6 ; Serpina5 ; Prss2 ; AF366264 ; Erap1 ; Serpina11 ; Bid ; Cst11 ; P2rx1 ; Acvr1c ; Dpep2 ; Cd27 ; Usp43 ; Sox7 ; Tmprss5 ; Itih1 ; Serpina3k ; Usp46 ; Mcpt9 ; Klk15 ; Gzmn ; Klk1b27 ; Tmprss11a ; Mmp9 ; St14 ; Robo1 ; Psmb11 ; Agbl2 ; Cd109 ; Akt1 ; Cma1 ; Mmp14 ; Ift57 ; Alg13 ; Xdh ; Usp18 ; Foxl2 ; Casp3 ; Ggt6 ; Src ; Cfb ; Spink11 ; Klk1 ; Serpinb3c ; Tmprss4 ; Bcl2a1d ; Umodl1 ; Spink12 ; Spink8 ; Sox2 ; Lap3 ; Zranb1 ; Pi16 ; 2010111I01Rik ; Ctla2a ; Adamts15 ; Serpina1f ; Ngp ; Psmb8 ; Hc ; Spink4 ; Gzmk ; Adamts9 ; Ctsk ; Usp49 ; 4930486L24Rik ; Mmp23 ; Wfikkn2 ; 8030411F24Rik ; Prss27 ; Klk7 ; Klk1b16 ; Ggt1 ; Sfrp2 ; Plau ; Crbn ; Pcsk1n ; Nrip3 ; Pycard ; Cst3 ; Ctsr ; Mbtps2 ; Pip ; Cck ; Tmprss11b ; Serpinb9c ; 2010005H15Rik ; Adamts20 ; Psen2 ; Asrgl1 ; Cdh1 ; Adam24 ; Ctsd ; Nek5 |
| **REGULATION OF CALCIUM-MEDIATED SIGNALING** | 9 | 0 | 2 | 25 | Kdr ; Hap1 ; Gstm7 ; Neurod2 ; Cd24a ; Rgn ; Actn3 ; Fkbp1a ; P2rx4 ; Igf1 ; Atp2b4 ; Cd4 ; Rcan1 ; Itpr1 ; Lmcd1 ; Cd3e ; Sla2 ; Cmya5 ; Akap6 ; Myo5a ; Ccl4 ; Jsrp1 ; Zap70 ; Adora3 ; Rcan2 ; Jph2 ; Tmem100 ; Nrg1 ; Lhcgr ; Slc12a1 ; Dmd ; Trat1 ; Cd8a ; Gpr143 ; Fcer1a ; Casq2 ; Trem2 ; Drd4 ; Cdh13 ; Pln ; Ada |
| **REGULATION OF HEART CONTRACTION** | 4 | 4 | 8 | 56 | Dsp ; Tnni3k ; Hopx ; Tac1 ; Myh7 ; Cxadr ; Sema3a ; Pde5a ; Pde4b ; Edn1 ; P2rx4 ; Atp2b4 ; Gja5 ; Bves ; Myh6 ; Prkg1 ; Scn5a ; Drd2 ; Slc8a1 ; Crhr2 ; Cav1 ; Hey2 ; Adora1 ; Cacna1d ; Irx5 ; Ryr2 ; Hcn2 ; Tgfb2 ; Thra ; Prkca ; Dsc2 ; Chga ; Epas1 ; Akap6 ; Kcne1l ; Nppa ; S100a1 ; Cacna1b ; Calca ; Stc1 ; Kcnq1 ; Csrp3 ; Dmpk ; Atp1a2 ; Adrb1 ; Cacna1c ; Avpr1a ; Hcn4 ; Bmp10 ; Ifng ; Gaa ; Slc1a1 ; Sumo1 ; Apln ; Scn3b ; Adra1d ; Pmch ; Uts2 ; Ctnna3 ; Edn2 ; Thrb ; Dmd ; Cacna1e ; Jup ; Trpm4 ; Glp1r ; Casq2 ; Nos3 ; Nos1 ; Cav3 ; Rgs2 ; Nup155 ; Agtr2 ; Edn3 ; Cacna1g ; Sp4 ; Npff ; Kcna5 ; Oxt ; Snta1 ; Rnf207 ; Chrna7 ; Ucn ; Mdm4 ; Pln ; Adra1b ; Ada ; Smad7 ; Zc3h12a ; Th ; Myl3 ; Kcne2 ; Fxyd1 ; Scn10a |
| **REGULATION OF LEUKOCYTE MEDIATED IMMUNITY** | 3 | 6 | 1 | 44 | Adora2b ; Fcer1g ; Cd24a ; Fcgr3 ; Pik3r6 ; Ung ; Malt1 ; Traf6 ; Rsad2 ; Vav1 ; C3 ; Clec4g ; Was ; Tlr4 ; Unc13d ; Zap70 ; Zbtb1 ; Clec2d ; Cd28 ; Nod2 ; Raet1b ; Il21 ; F2rl1 ; Adora3 ; Il7r ; Lep ; Hpx ; Lta ; Rasgrp1 ; Fcer2a ; Il4 ; Tap1 ; Serpinb9 ; Ifnb1 ; Ifng ; Gab2 ; Tnfsf4 ; Lyn ; Cd84 ; Il23a ; Nlrp3 ; Sh2d1b2 ; Ptprc ; Fgr ; Slamf1 ; Stat5b ; Fzd5 ; H60a ; Klre1 ; Serpinb3a ; P2rx7 ; Spn ; Il4ra ; Fcgr2b ; Ms4a2 ; Ulbp1 ; Tlr3 ; Il13ra2 ; Cd226 ; Trpm4 ; Xcl1 ; Tnf ; Sh2d1b1 ; Klrb1b ; Ccr2 ; Fcer1a ; Clcf1 ; Lag3 ; Muc4 ; Slamf6 ; Klrk1 ; Crtam ; Bcl6 ; Crhr1 ; Tap2 ; Cd1d2 ; H2-M3 ; Ptafr ; Paxip1 ; Fcgr1 ; Tbx21 |
| **REGULATION OF LYMPHOCYTE MEDIATED IMMUNITY** | 2 | 3 | 1 | 35 | Fcer1g ; Cd24a ; Fcgr3 ; Pik3r6 ; Ung ; Malt1 ; Traf6 ; Rsad2 ; Vav1 ; C3 ; Clec4g ; Was ; Zbtb1 ; Clec2d ; Cd28 ; Nod2 ; Raet1b ; Il21 ; Il7r ; Lep ; Hpx ; Lta ; Rasgrp1 ; Fcer2a ; Il4 ; Tap1 ; Serpinb9 ; Ifnb1 ; Ifng ; Tnfsf4 ; Il23a ; Nlrp3 ; Sh2d1b2 ; Ptprc ; Slamf1 ; Stat5b ; Fzd5 ; H60a ; Klre1 ; Serpinb3a ; P2rx7 ; Spn ; Fcgr2b ; Ulbp1 ; Cd226 ; Trpm4 ; Xcl1 ; Tnf ; Sh2d1b1 ; Klrb1b ; Fcer1a ; Clcf1 ; Lag3 ; Muc4 ; Slamf6 ; Klrk1 ; Crtam ; Bcl6 ; Tap2 ; Cd1d2 ; H2-M3 ; Paxip1 ; Fcgr1 ; Tbx21 |
| **TRANSFORMING GROWTH FACTOR BETA RECEPTOR SIGNALING PATHWAY** | 10 | 10 | 6 | 22 | Ski ; Folr1 ; Tdgf1 ; Nepn ; Aspn ; Fos ; Foxh1 ; Onecut2 ; Bmpr1b ; Htra1 ; Prdm16 ; Tgfbr2 ; Myocd ; Cdkn2b ; Adamtsl2 ; Cdh5 ; Cited1 ; Hpgd ; Cav1 ; Nkx2-1 ; Col1a2 ; Dusp15 ; Hipk2 ; Cav2 ; Bmp2 ; Nodal ; Tgfb2 ; Htra3 ; Wnt1 ; Tgfbr3 ; Smurf1 ; Chst11 ; Fbn1 ; Lrg1 ; Trp53 ; Gdf2 ; Thbs1 ; Usp15 ; Ptprk ; Mstn ; Bambi ; Il17f ; Usp9x ; Ltbp1 ; Dand5 ; Ltbp3 ; Itgb5 ; Cav3 ; Acvr1c ; Cd109 ; Zfyve9 ; Src ; Acvrl1 ; Npnt ; Wfikkn2 ; Gdnf ; Tgfb3 ; Smad7 |
